# A bottom-up approach to find lead compounds in expansive chemical spaces

**DOI:** 10.1038/s42004-025-01610-2

**Published:** 2025-08-01

**Authors:** Álvaro Serrano-Morrás, Andrea Bertran-Mostazo, Marina Miñarro-Lleonar, Arnau Comajuncosa-Creus, Adrià Cabello, Carme Labranya, Carmen Escudero, Tian V. Tian, Inna Khutorianska, Dmytro S. Radchenko, Yurii S. Moroz, Lucas Defelipe, David Ruiz-Carrillo, Maria Garcia-Alai, Robert Schmidt, Matthias Rarey, Patrick Aloy, Carles Galdeano, Jordi Juárez-Jiménez, Xavier Barril

**Affiliations:** 1 Unitat de Fisicoquímica, Departament de Farmàcia i Tecnologia Farmacéutica, i Fisicoquímica. Facultat de Farmàcia i Ciències de l’Alimentació, Universitat de Barcelona (UB), Av. Joan XXIII, 27-31, 08028 Barcelona, Spain; 2 Institut de Biomedicina, Facultat de Biologia, Universitat de Barcelona (UB), Av. Diagonal, 643, 08028 Barcelona, Spain; 3 Institut de Recerca Biomèdica (IRB Barcelona) and Barcelona Institute of Science and Technology (BIST). c/ Baldiri i Reixac, C\ Baldiri i Reixac, 10-12, 08028 Barcelona, Spain; 4 Upper Gastrointestinal and Endocrine Tumor Group, Vall d’Hebron Institute of Oncology (VHIO), Vall d’Hebron Barcelona Hospital Campus, C/ Nazaret 115-117, 08035 Barcelona, Spain; 5 Enamine Ltd., 78 Winston Churchill St., 02094 Kyїv, Ukraine; 6 Chemspace LLC, 85 Winston Churchill St., 02094 Kyїv, Ukraine; 7 Taras Shevchenko National University of Kyiv, 60 Volodymyrska St, 01601 Kyїv, Ukraine; 8 European Molecular Biology Laboratory, Building 25a, DESY, 22607 Hamburg, Germany; 9 Centre for Structural Systems Biology (CSSB), Building 15, DESY, 22607 Hamburg, Germany; 10 Center for Bioinformatics, University of Hamburg ZBH, Albert-Einstein-Ring 8-10, 22761 Hamburg, Germany; 11 Catalan Institution for Research and Advanced Studies (ICREA), Pg. Lluis Companys 23, 08010 Barcelona, Spain; 12 Institut de Química Teòrica i Computacional (IQTC), Facultat de Química i Física, Universitat de Barcelona (UB), C. Martí i Franqués, 1, 08028 Barcelona, Spain; 13 Present Address: BioSolveIT GmbH, An der Ziegelei 79, 53757 St.Augustin, Germany

**Keywords:** Computational chemistry, Biophysical chemistry, Cheminformatics, Virtual screening

## Abstract

Drug discovery starts with the identification of a “hit” compound that, following a long and expensive optimization process, evolves into a drug candidate. Bigger screening collections increase the odds of finding more and better hits. For this reason, large pharmaceutical companies have invested heavily in high-throughput screening (HTS) collections that can contain several million compounds. However, this figure pales in comparison with the emergent on-demand chemical collections, which have recently reached the trillion scale. These chemical collections are potentially transformative for drug discovery, as they could deliver many diverse and high-quality hits, even reaching lead-like starting points. But first, it will be necessary to develop computational tools capable of efficiently navigating such massive virtual collections. To address this challenge, we have conceived an innovative strategy that explores the chemical universe from the bottom up, performing a systematic search on the fragment space (exploration phase), to then mine the most promising areas of on-demand collections (exploitation phase). Using a hierarchy of increasingly sophisticated computational methods to remove false positives, we maximize the success probability and minimize the overall computational cost. A basic implementation of the concept has enabled us to validate the strategy prospectively, allowing the identification of new BRD4 (BD1) binders with potencies comparable to stablished drug candidates.

## Abstract (web summary)

The vast scale of emerging on-demand chemical collections presents a challenge for efficiently identifying promising drug candidates. Here, the authors develop a bottom-up computational strategy to first explore fragment space and then exploit the most promising scaffolds, successfully identifying diverse and potent BRD4 binders.

## Introduction

Ultra-large compound combinatorial spaces are becoming an essential component of the drug-discovery pipeline. While up to the mid-2010s the average size of chemical libraries typically used in drug-discovery efforts oscillated between 10^5^ and 10^6^ compounds, the development of billion-sized on-demand compound collections has significantly expanded the chemical matter available for drug-discovery efforts. As long as they maintain a healthy chemical diversity^1^, these ultra-large collections can increase the probability of identifying drug-like high-affinity ligands. However, the computational effort of enumerating, preparing, navigating, and evaluating these expansive chemical spaces quickly becomes prohibitive. Within the last decade, there have been major advances in the scale and success of high-throughput virtual screening (VS) campaigns^2–5^, but brute-forcing the docking calculations of the whole, enumerated, ultra-large collections is not scalable nor sustainable with their current growth rate^6^. Therefore, it is imperative to develop new approaches that enable the efficient exploration of chemical space, whose cost does not escalate with the predicted growth of commercial on-demand chemical spaces.

Recent developments in hardware and machine learning (ML) algorithms have enabled the application of active learning (AL) based approaches to speed up VS campaigns using ultra-large compound collections. Most of the reported approaches use ML-accelerated docking^7–10^ that has proven useful to identify hit compounds, but it remains heavily reliant on the accuracy of the underlying docking scoring function and requires the enumeration of large regions of the chemical space into slices of size akin to the traditional VS screening libraries. A potential alternative is offered by generative models^11,12^, but nowadays these models still frequently propose small molecule compounds that are not synthetically feasible, with unreliable binding poses with incorrect topologies (e.g., wrong bond lengths and angles, torsional strains)^13^, nor improve the overall accuracy of docking methods^14^.

**Fig. 1:**
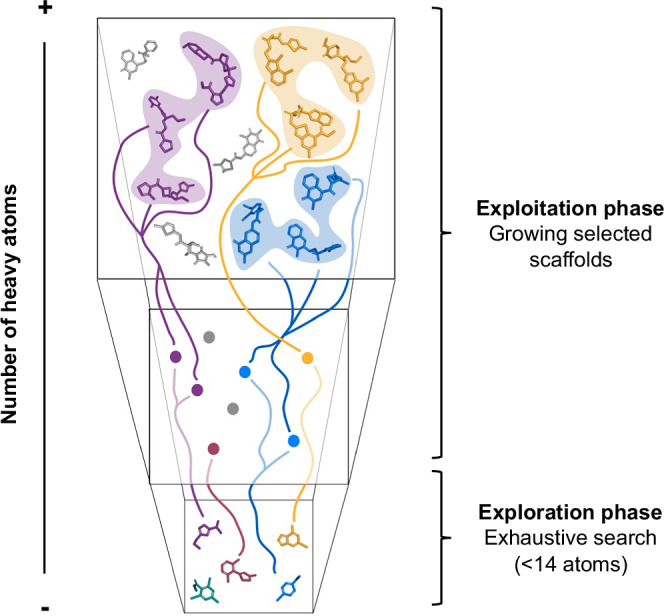
Schematic representation of a bottom-up approach for the exploration of vast chemical spaces. The low molecular weight space (bottom) has fewer possible molecules and can be explored systematically. Promising hits in this space signal privileged areas of chemical space that are exploited by enumerating scaffold-focused libraries (colored lines) from vast on-demand collections. Virtual screening of these focused collections leads to the identification of potent drug-like compounds.

An appealing alternative to the exhaustive screening of ultra-large chemical collections would be to focus the efforts in exploring and evaluating only the regions of the chemical space more likely to contain compounds able to bind to a given protein of interest^15^. Synthons-based approaches^16–20^ exploit the ultra-large combinatorial collections’ architecture to limit the compound’s enumeration by independently docking synthetic building blocks (or synthons) and subsequently growing them in a synthon-step manner, which is constrained by the reaction pool encoded in the collection. In this work we combine the strengths of exhaustive exploration and synthon-based strategies, leveraging natural properties of chemical space (exponential growth with number of atoms and recursive use of the same components) to propose a bottom-up approach that can be applied to any chemical collection regardless of their composition (Fig. 1). The exploration phase of the approach focuses on the “bottom” of the chemical space: fragment-sized compounds suitable for medicinal chemistry (ca. 10^9^ compounds containing up to 14 heavy atoms)^21^. This region constitutes a complete but relatively small fraction of the overall chemical space and, therefore, we can exhaustively explore it with the current technological capabilities and identify low molecular-weight compounds with high ligand efficiencies^22^. The fragment hits are then analyzed to define the essential core for target binding (specific scaffolds or more abstract substructures), which we can use to query chemical spaces, enumerating comprehensive focused libraries at “upper” layers of the chemical space that are systematically explored to define a short list of candidates (exploitation phase). Importantly, we analyze the compounds through a hierarchy of increasingly sophisticated computational methods, from molecular docking to molecular dynamics (MD) based methods. By trading speed for accuracy at each step, we can apply incrementally more accurate methods to a smaller number of compounds. We demonstrate that this innovative approach, which can be fully automatized, vastly reduces the fraction of chemical space to be evaluated while delivering a very high hit rate.

To provide proof-of-concept, we have applied this bottom-up approach to identify compounds targeting the first repeat of the Bromodomain and Extra-Terminal domain 4, BRD4 (BD1), whose role in cell proliferation, apoptosis, and transcription has made it a very appealing target^23^. Additionally, the wealth of information available for BRD4 (BD1) allowed us to demonstrate the applicability and potential of our approach in different situations that are common starting points in new drug discovery programs. The first validation scenario corresponds to a direct lead discovery process in which there would not be prior information about chemical matter binding to our target of interest. Thus, we would need to apply the complete protocol, from a druggability assessment of the binding site, to prioritizing scaffold hits from an exhaustive fragment screening, and then the expansion of the chosen scaffolds using the ultra-large chemical spaces. The second scenario recreates a situation where the binding mode of fragments or drug-like compounds to the target of interest is known, but we seek increased potency or chemical novelty to advance to the lead stage. This would only require the scaffold expansion section of the bottom-up approach. We have evaluated this second scenario using (a) starting points from crystallized fragments available in the Protein Data Bank (PDBids: 4LZS, 6ZED, 6ZF9), where the optimization vectors are unknown, and (b) from chemical scaffolds extracted from advanced drugs ((+)−JQ1^24^, IBET-151^25^ and ABB-V075^26^), to validate the approach obtaining a diverse set of *me-too* or *me-better* compounds, simulating also a common hit-to-lead situation.

We demonstrate the power of this computational approach by identifying and experimentally validating hits in the different scenarios, with a success rate close to 20% in each case, achieving great diversity in the chemical space identified.

## Results

### A hierarchal computational approach to explore vast chemical spaces

**Fig. 2:**
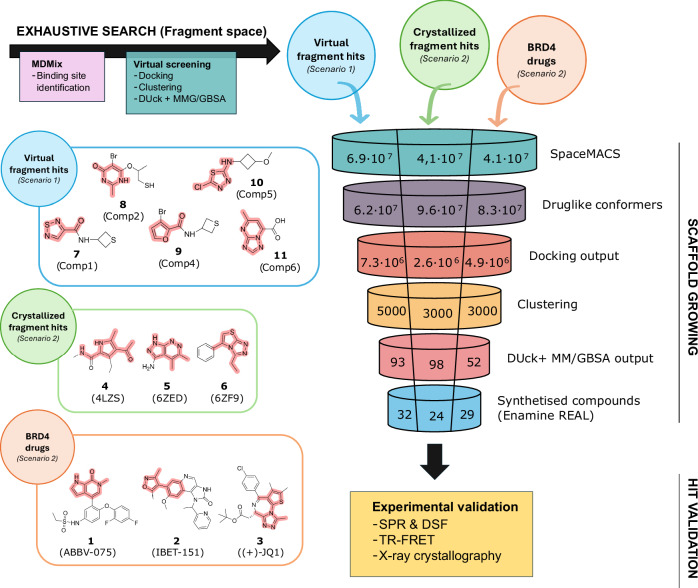
Schematic representation of the workflow presented for the exploration of the ultra-large compound collections. Given a no-previous knowledge scenario (Scenario 1; blue), virtual fragment hits are identified using a fragment-based virtual screening approach driven by MDMix-found pharmacophores. If initial binders are known (Scenario 2; green and red), the main core scaffolds are derived. The initial fragments and scaffolds are highlighted for each scenario (left). Selected scaffolds are grown by creating scaffold-focused libraries, which, after prepared and docked, are clustered to be evaluated using a combination of DUck and MM/GBSA. Selected compounds are then synthesized and tested experimentally. The values correspond to the number of molecules evaluated at each stage, per scenario.

Our bottom-up approach for the exploration of ultra-large chemical spaces is basically divided in two stages (Fig. 2a): (i) an exhaustive exploration of the low molecular weight chemical space to identify fragment-sized compounds, (ii) the growth of those fragment-sized compounds into drug-sized compounds with higher affinity and drug-like properties. For these stages, different computational methods are applied following a hierarchy based on the tradeoff between accuracy and throughput, only progressing the highest-scored compounds by each method to the next layer. By following this hierarchical approach, we reduce the number of candidate molecules to be considered, and thus the overall resources allocated to sampling less-promising areas of the chemical space, which allows us to use computationally more demanding methods without significantly affecting the overall throughput. Specifically, the approach relies on docking to obtain a prediction of the binding mode for each compound, followed by clustering and a diversity analysis of the top-ranked compounds. Subsequently, the Molecular Mechanics—Generalized Born Surface Area method (MM/GBSA)^27^ is used to rank the molecules by solvation energy, and the 1000 molecules with the lower predicted desolvation penalty are further assessed by dynamic undocking (DUck)^28^. A consensus of the MM/GBSA and DUck results is then used to prioritize the compounds to be biophysically validated. The experimental validation for the approach was pursued orthogonally in a three-step manner: first, the compounds were assessed with a double single-dose screening by Differential Scanning Fluorimetry (DSF) and Surface Plasmon Resonance (SPR). Second, we also sought to screen and experimentally confirm the predicted binding mode of the selected ligands by X-ray crystallography. Lastly, quantitative binding affinities are obtained by dose-response testing in a competitive Time-Resolved Förster Energy Transfer (TR-FRET) based assay.

### Discovery of novel BRD4 (BD1) compounds

The implementation of the approach in the aforementioned stages makes it highly versatile, as it can be initiated at any point of the workflow, either from the bottom, with an exhaustive virtual fragment screening as in the first scenario, or directly in the scaffold growing step, like in the second scenario. The first scenario (hereafter denoted as the *virtual fragment hits*), which recreates a scenario where there would not be prior knowledge of compounds interacting with our target, requires identifying the interaction hotspots in the binding site using MDMix^29^ (Fig. S1). MDMix simulations of BRD4 (BD1) resulted in the identification of a polar interaction with the Nδ atom of Asn140 and a hydrophobic hotspot in the vicinity of a cluster of water molecules at the bottom of the BRD4 (BD1) binding site. We used these hotspots as pharmacophoric restraints while docking the fragment collection (ca. 4 M unique fragments obtained from Enamine REAL^30^ database and ZINC20^31^). This resulted in 3.5 10^8^ conformers that complied with both pharmacophoric restraints and obtained favorable docking scores. These fragments were then grouped using the Chemical Checker *signaturizers* (CCS)^32^ into 2000 clusters, and we used MM/GBSA to filter out those clusters whose representative compound was estimated to bind with ΔG_bind_ > −30.0 kcal/mol. This procedure yielded homogenous clusters, and an analysis of their representative compounds revealed that it also maximized the recovered chemical diversity of the fragment library (Fig. S8.b–d). We used DUck to screen the remaining 973 fragments. This MD-based method measures the work needed to reach a quasi-bound state (W_QB_) where a key protein-ligand interaction (in this case the hydrogen bond with Asn140) is broken. Setting a W_QB_ threshold of 7.0 kcal/mol, we obtained five fragments **7**, **8**, **9**, **10**, and **11** (virtual fragment hits, Fig. 2b) containing unique scaffolds. We used these five fragments as the starting point for the scaffold growing stage of the pipeline, where we aimed to obtain potent drug-sized compounds. To this end, we used SpaceMACS^33^ to search for drug-sized compounds containing the corresponding scaffolds in the REAL Space ultra-large database. We set a maximum of 20 million compounds per scaffold, which was deemed a reasonable balance between exploration, exploitation, throughput, and hardware requirements. These scaffold-focused libraries were then filtered, excluding non-drug-like molecules (solubility, rotatable bonds and Ro5 considered), and prepared according to VS standard procedures, generating the appropriate tautomers, protomers and alternate ring conformations, which yielded libraries containing 16 M, 27 M, 16 M, 300.000 and 2.8 M compounds per scaffold respectively (See Methods Section and Table S2 for details). Subsequently, we docked the compounds, restraining the position of atoms belonging to the scaffolds (thus preserving their position from the parent fragment) using the tethered docking implementation in rDock^34^. The tethered docking stage filtered between 81% and 99% of the compounds of each library, leaving 2.7 M, 3.8 M, 220.000, 2.400, and 530.000 compounds derived from the five scaffolds, respectively. We grouped the docked compounds using the CCS into 1000 clusters per scaffold, each containing between 1 and 600 molecules (with a median cluster size of 250 molecules, Fig. S8.a). We used MM/GBSA and DUck to obtain a final ranking of the cluster representative compounds, excluding the compounds with predicted desolvation penalties higher than 20 kcal/mol and all the compounds that obtained a W_QB_ lower than their parent fragment. The threshold values applied at each filtering stage were selected based on the throughput available for the following step, and are susceptible to be changed depending on the available computational resources. The top ten compounds from each scaffold, ranked according to a consensus score from the MM/GBSA’s ΔG_bind_ and DUck’s W_QB_, were ordered to be synthesized by Enamine and were validated biophysically.

In the second scenario, we leveraged a handful of crystallographic structures to directly start the scaffold growing stage from an already corroborated binding pose. Depending on the discovery stage of the crystallized compounds we classified (i) the fragment-sized molecules that had not undergone further fragment growing strategy as the *crystallized fragment hits* case, (ii) and the optimized BRD4 (BD1) compounds as the *BRD4 drugs* case. We selected the crystallized fragments in the 4LZS (*K*d = 6.8 µM)^35^, 6ZED (IC_50_ = 72 µM)^36^ and 6ZF9 (IC_50_ = 26 µM)^36^ PDB entries for the first case, and the advanced BRD4 (BD1) drugs ABBV-075 (*K*_i_ = 11 nM)^26^, IBET-151 (IC_50_ = 0.79 µM)^25^ and (+)−JQ1 (IC_50_ = 77 nM)^24^ for the second case. The first step was to derive the essential scaffolds of the crystallized fragment and drugs, maintaining their core pharmacophoric features. To grow these starting points into drug-like compounds from the ultra-large collections, we applied the same workflow as in the previous scenario. In the *BRD4 drugs* case, we employed a more permissive SMARTS^37^ encoding of the scaffolds in order to recover substructure matches for the more complex compounds (Table S1). The resulting scaffold-focused libraries were then filtered by drug-likeness, obtaining between 10 M and 16 M compounds per scaffold (Table S2), which approximately doubled in size after considering different ring conformations, protonation, and tautomerization states. After the tethered docking process, we obtained binding poses for 5% to 20% of the compounds, depending on the scaffolds, due to the reduced number of rotational and translational degrees of freedom imposed by the tethered docking approach, which led to many compounds predicted to produce steric clashes with the protein. Furthermore, based on the score value of the parent compounds (SCORE.INTER ca. -10), we filtered out those compounds that did not improve that threshold. Then, after clustering these libraries using CCS, we used MM/GBSA and DUck to obtain a final ranking of the compounds, excluding those with high desolvation penalties and a low W_QB_ as done for the first scenario (Table S2). Using the consensus scoring, the top ten ranked compounds for each scaffold-focused library were ordered to be synthesized by Enamine and validated experimentally as in the first scenario. The outcome of applying our bottom-up approach to these two hypothetical scenarios is summarized in Table 1.

**Table 1:** Summary of the resulting molecules at each step of the computational pipeline

	Scenario 1	Scenario 2	
Scaffold source	Virtual fragment hits	Crystallized fragment hits	BRD4 drugs
Number of cpds. resulting from substructure search	6.84 × 10^7^	4.10 × 10^7^	4.10 × 10^7^
Number of drug-like 3D conformers	6.36 × 10^7^	10^8^	8.3 × 10^7^
Successfully docked structures	7.25 × 10^6^	2.58 × 10^6^	4.9 × 10^6^
Number of clusters (1000 clusters per scaffold)	5000	3000	3000
Cpds. fulfilling MM/GBSA and DUck consensus threshold(1 compound evaluated per cluster)	93	98	52
Synthesized cpds. (from the top 10 scored)	32 (out of 42)	24 (out of 30)	29 (out of 30)

### Biophysical validation of the BRD4 (BD1) compounds discovered

**Fig. 3:**
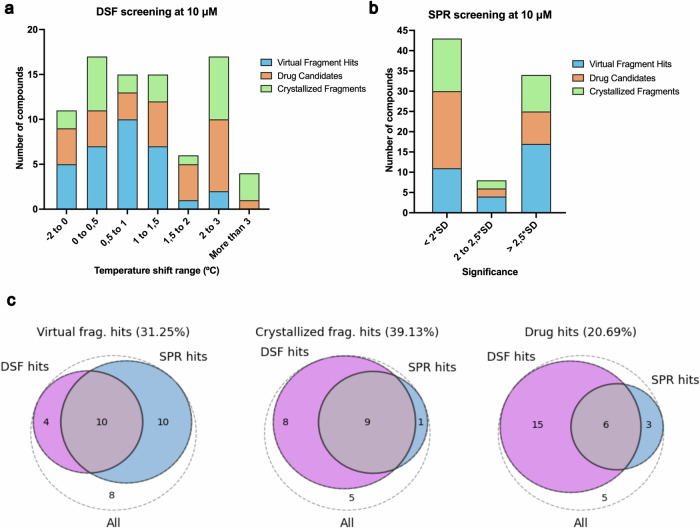
Results of the initial screening by DSF and SPR. **a** Results of the single dose screening at 10 µM by DSF showing the number of compounds grouped by the degrees of thermal shift of BRD4 (BD1). **b** Results of the single dose screening at 10 µM by SPR showing the number of compounds grouped by the significance obtained compared to the average of the blanks. **c** Venn diagrams combining the results obtained by DSF and SPR grouped by the different initial scenarios.

To experimentally validate the developed bottom-up approach, we characterized the binding of the identified compounds using biophysical assays. In total, we selected 102 compounds to be synthesized, of which 85 (83%) were successfully synthesized and delivered by Enamine (Fig. S9). First, we conducted a double orthogonal single-dose screening at 10 µM by DSF and SPR, using (+)-JQ1 as positive control (Fig. 3). On the one hand, in the DSF screening, we determined the melting temperature of the apo BRD4 (BD1) to be 46,36 ± 0,26 °C (*n* = 3), and hence established a deviation in the melting temperature of 0.53 °C (twice the standard deviation) as a threshold to consider a compound as a positive binder in the screening. We screened all the synthesized compounds at 10 µM concentration by triplicate (*n* = 3). Using this criterion, a total of 52 compounds were considered as DSF hits, 14 compounds obtained from the *virtual fragment scaffolds* (44% hit rate), 17 compounds from the *experimental fragment scaffolds* (71% hit rate), and 21 from the *BRD4 drug scaffolds* (72% hit rate) (Fig. 3a, and Supplementary Data 1). In parallel, compounds were considered as SPR hits also when their average signal was further than two standard deviations apart from the average signal of the blank samples (1% DMSO). Doing this, we ensured that the signal in the SPR was exclusively due to the compound binding and not due to the DMSO used to solubilize the compounds, as it has been reported to bind BET domains^38^. We screened by SPR all the compounds synthesized at 10 µM by duplicate (*n* = 2). Using this requirement, we respectively considered 20 (63%), 10 (42%), and 9 (31%) compounds as SPR hits from each validation case, respectively, (Fig. 3b and Supplementary Data 1). Combining data from both single-dose screenings (Fig. 3c), 25 compounds resulted positive in both DSF and SPR techniques and were selected for further experimental validation.

**Fig. 4:**
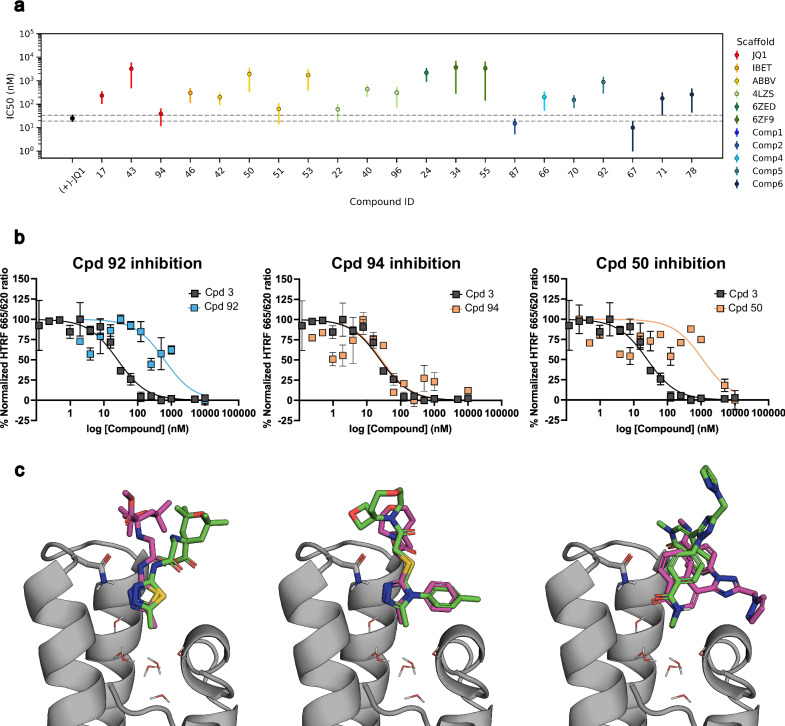
Results of HTRF TR-FRET and X-ray experiments. **a** Summary of the IC_50_ values obtained by HTRF TR-FRET for the DSF/SPR hits and X-ray screening hits. Error bars correspond to the 95% confidence interval (*n* = 2) **b** Examples of the dose-response curves assessed by HTRF TR-FRET, comparing the (+)−JQ1 control (compound 3, black) and three example compounds (blue and orange). Each dot represents the average of two independent replicas, and the error bars correspond to their standard deviation. **c** X-ray structures of compounds 92 (Supplementary Data 2; PDB 9HT2), 94(Supplementary Data 3; PDB 9HT1), 50 (Supplementary Data 4; PDB 9HT0), bound to BRD4 (BD1). In the picture are illustrated the predicted binding pose (pink) and the resolved crystallographic structure (green).

To further quantitatively characterize the DSF/SPR hits compounds obtained in the single-dose screenings, we selected HTRF TR-FRET. Specifically, we employed a competitive HTRF TR-FRET experiment to measure the compound’s capacity to displace an acetylated peptide from the binding site of BRD4 (BD1). For each molecule, a 16-point concentration curve was titrated against the peptide-BRD (BD1) complex and incubated for 1.5 h. A compound was considered a positive binder if we could fit its titration curve to a sigmoidal curve to determine its IC_50._ We determined IC_50_ values (Fig. 4a) ranging from low nM (compounds **67**, **87**, and **94**) to one- or two-digit μM (compounds **24**, **43**, **92**). In the end, 19 BRD4 (BD1) binders (Supplementary Data 1) from the 25 DSF/SPR hits showed a dose-response behavior in the TR-FRET experiments. Of the 19 validated binders, 7 were identified from the *virtual fragment hits*, 6 were derived from *BRD4 drugs*, and 6 were derived from the *crystallized fragments* case. To obtain additional validation of our bottom-up approach, we also screened the 85 compounds purchased via X-ray crystallography. These could provide additional positive compounds and an additional layer of validation of the binding modes proposed at the docking stage. We were able to obtain compounds **50**, **92**, and **94** bound to of BRD4 (BD1) (Fig. 4c). None of these compounds were considered DSF/SPR positive hits. Nonetheless, the corresponding IC_50_ values were obtained by TR-FRET (Fig. 4b). X-ray diffraction pattern analysis revealed that compounds **92** (IC_50_ = 621.8 nM) and **94** (IC_50_ = 27.9 nM) bound with the expected binding mode based on the docking calculations. Compound **50**, which is a weak binder (IC_50_ = 1129 nM) derived from drug **1** (ABBV-075) with known binding mode, surprisingly showed engagement with BRD4 in an upside-down pose with respect to the parent compound but retained the pharmacophoric features observed for the remaining compounds.

### The bottom-up approach developed provides chemical diversity

**Fig. 5:**
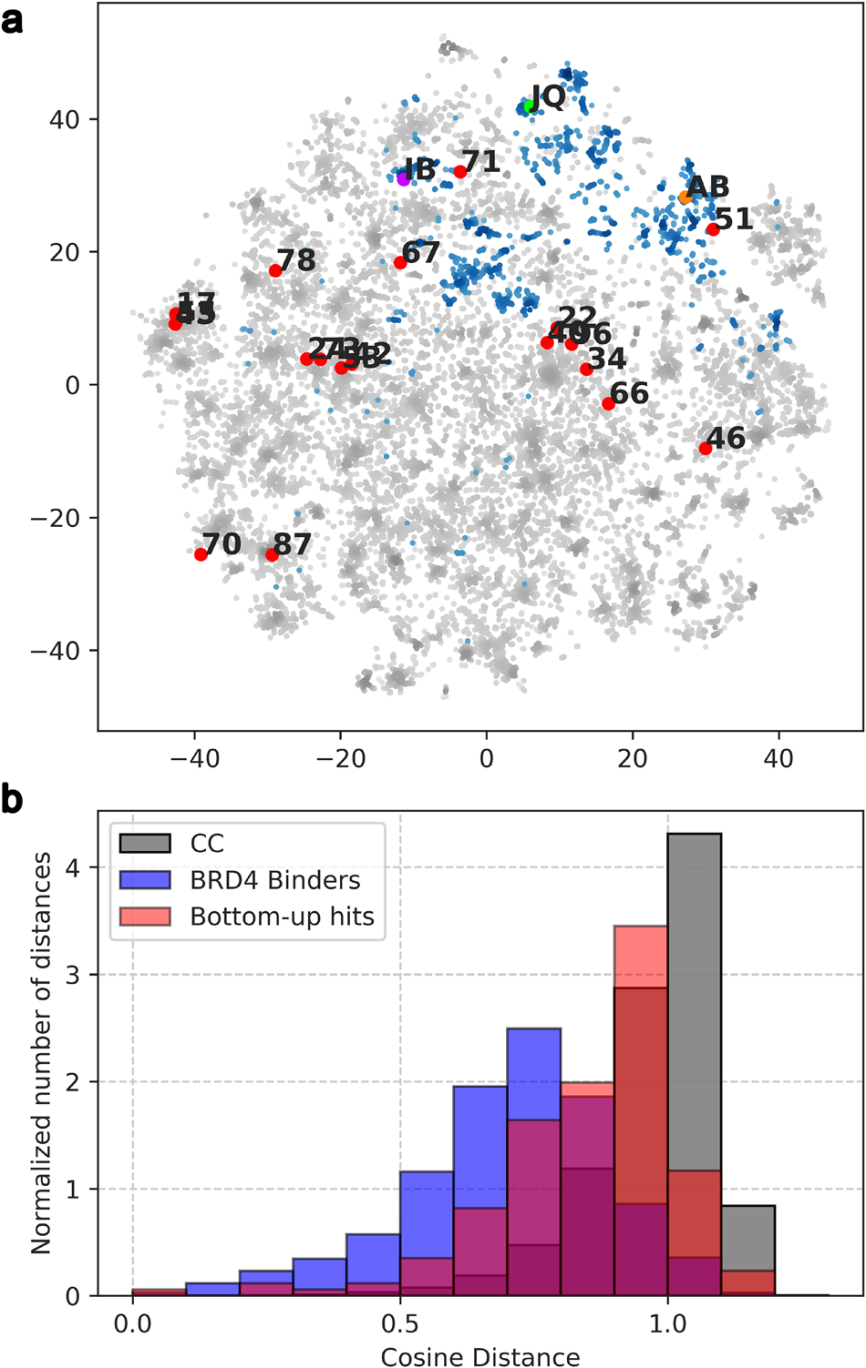
Analysis of the chemical diversity of the most potent compounds compared to the described BRD4 (BD1) binders. **a** Illustration of the chemical diversity. Representation of the random molecules from Chemical Checker chemical space (gray), known BRD4 (BD1) binders from ChEMBL (blue), and validated binder compounds discovered (red). **b** Histogram exhibiting the intragroup distance’s distribution (cosine distance) for compounds obtained from the bottom-up approach (orange), a set of randomly selected compounds from the Chemical Checker chemical space (green), and the known BRD4 binders (blue).

At this point, we wanted to assess whether the bottom-up approach was simply identifying closely related derivatives of compounds known to bind to BRD4 (BD1) or if, as we aimed, it could be a useful tool to explore new regions of the chemical space. To this end, we compared the chemical diversity of the 19 validated binders with that of a random sample of the Chemical Checkers universe^32^ and all the BRD4 (BD1) binders described in ChEMBL^39^ (Fig. 5). The 19 binders were sparsely distributed among the chemical diversity represented in the Chemical Checkers and did not cluster near the known BRD4 (BD1) binders. Furthermore, the distribution of cosine distances between pairs of compounds resembled more the one calculated for the randomly selected compounds than that of known BRD4 (BD1) binders, which indicates that the identified compounds are chemically diverse and occupy distinct regions of chemical space.

In summary, we demonstrated that our bottom-up approach was able to identify novel compounds in two common use cases in the pharmaceutical industry, identifying and validating by biophysical assays 19 novel lead-potency compounds binding to BRD4 (BD1). These compounds are potent (IC_50_s up to the low nM range by HTRF TR-FRET) and chemically diverse, representing good starting points for lead optimization campaigns.

## Discussion

Ultra-large on-demand combinatorial chemical spaces have become extremely popular and have the potential to substantially impact the field of drug discovery, providing more and better compounds to start lead optimization campaigns. However, their transformative potential relies on our ability to develop effective and efficient new ways to explore the chemical space. In this work, we have developed a computational approach based on: (a) exhaustive exploration of the low molecular-weight chemical space and subsequent generation of scaffold-focused libraries, and (b) the application of a hierarchy of computational methods to maximize the quality of the predictions. This approach shares similarities with the synthon-based strategies^16–18^, which exploit the graph architecture of the ultra-large chemical spaces to confine the enumeration around certain structural regions, greatly reducing the computational cost. However, by using SMARTS-based substructural queries, we avoid constraining the search to specific building blocks, therefore sidestepping the main limitation of synthon-based approaches. The encoding of substructures in SMARTS and the subsequent search with SpaceMACS allows us to control the scaffolds’ growth vectors, tailoring the outputs to the topological features of our protein of interest. Moreover, the substructures encoded in this manner can cover multiple building blocks, in contrast with the synthon definition, and thus enable a more efficient exploration of the chemical space neighboring each scaffold. These searches can be applied to all existing on-demand collections, regardless of their synthon composition or substructure complexity, which is crucial to explore those chemical spaces with an undisclosed synthon composition, whose providers consider it part of their intellectual property.

The proposed bottom-up approach relies on the identification of a minimal molecular core capable of binding to the target of interest in a defined binding mode. As we have demonstrated, this core can have several origins (in silico fragments hits discovered in this work, crystallized fragments, or known drug-like ligands), but knowledge of its binding mode is essential, as it will be preserved throughout the process. The working hypothesis of a stable binding mode is commonly used in the evaluation of congeneric compound series^40–42^. In addition, the resolved crystal structures of described fragments and compounds bound to BRD4 (BD1) confirmed this hypothesis (Fig. 4c) and validated the tethered approach employed. Tethering the binding mode during docking reduces the conformational space, enabling further computational speedup. In that regard, we emphasize the importance of using a battery of methods of increased accuracy and precision to overcome the limitations of docking calculations. Here we used MM/GBSA and DUck as they have shown good results complementing the rDock scoring function^36^, but due to the modularity of the approach, it can be easily extended to include other filtering methodologies. As an example, alchemical free energy transformations could be added to the pipeline, either as Free-Energy Perturbation (FEP) networks for the evaluation of the scaffold-focused libraries or Absolute Binding Free-Energy (ABFE) for the binding of the initial fragments.

In stark contrast to exhaustive exploration, a bottom-up approach is posed to be highly sustainable in time. Specifically, all the work described in this manuscript entailed the VS of a total of ca. 155 M compounds, which represented a mere 0.7% of Enamine REAL collection at the time. This exercise had a computational cost of ~3 × 10^4^ CPU hours, using HPC facilities. By comparison, using an average of 10 s per molecule in docking, evaluating an equivalent number of compounds through brute-force docking would take the order of 3 × 10^8^ CPU hours, without considering the time required for library enumeration. As such, by constraining the space to evaluate promising scaffolds, we have achieved a theoretical ten-thousand-fold reduction in the computational cost, even when evaluating the ligands through more sophisticated techniques than docking.

We prospectively validated the pipeline with the identification of novel BRD4 (BD1) binders from Enamine REAL Space (ca. 20 billion compounds at the time of this work). To fully assess the potential of the strategy, we explored two common scenarios when seeking new binders for any protein of interest, obtaining a combined total of 19 validated binders (22% hit rate), with potencies in the low micromolar to nanomolar range, comparable to fully developed BRD4 (BD1) drug candidates. The obtained hit rate and the potency of the identified hits showed a similar performance to other existing methods to explore ultra-large chemical spaces^43^. Interestingly, we obtained higher success rates from the simpler starting points: the in silico and *crystallized* fragment hits. We hypothesize that the complexity of the scaffolds found in optimized drugs (e.g., benzodiazepine scaffold of (+)−JQ1), which are little exampled in on-demand chemical collections, could account for the lower number of leads recovered for these scaffolds.

Moreover, all the validated binders showed a great chemical diversity and novelty compared to previously reported BRD4 binders, which have been thoroughly studied and targeted. Indeed, those identified hits which appear closer to the already known BRD4 binders (**51**, **71**, **73**) don’t necessarily come from the second scenario. This highlights the potential of exploiting these on-demand collections to open new intellectual property venues even in highly exploited targets, or to access more favorable scaffolds for lead optimization phases.

While massive collections are set to rapidly increase their size, based on the little overlap between vendors, future growth will mostly stem from an expansion of the covered chemical space rather than increasing the density of currently explored regions, which will make large increases in chemotype depth highly unlikely^6^. It is predicted that the depth around chemotypes will increase by 2-fold or 3-fold for every 10-fold increase in overall collection size. At that rate, approaches based on the identification of privileged regions of the chemical space and exhaustive exploration of scaffold-focused libraries, like the one presented here, will remain effective in the years to come.

## Materials and methods

### Receptor and compound library preparation

The PDB structure of BRD4 (BD1) in complex with 3-methyl-4phenyl-1,2-ozazol-5-amine (PDB code 4LR6) was prepared with the Molecular Operating Environment v2020-2^44^ using standard amino acid residues protonation states at pH 7.0. The co-crystallized small molecule was removed, and seven structural water molecules in the binding site were retained for the docking stage and subsequent calculations.

The low-molecular-weight library was built using two different compound collections, Enamine REAL database and ZINC20 (up to 350 MW). Both collections were filtered using RDKit^45^, selecting compounds with 14 or fewer heavy atoms and compounds containing at least one ring. After removing duplicates between both databases, the low-weight chemical collection contained ca. 4 million unique SMILES. Subsequently, protonation and tautomerization states were generated using Jchem v.20.21.0 (ChemAxon^46^) and Corina (v. 4.4.0)^47^ was used to generate up to four stereoisomers and up to five ring conformations, adding the required missing H atoms in the process. The final low-weight library contained ca. 12 million molecular structures to be docked.

To generate the scaffold-focused libraries, an early developmental version of SpaceMACS (v0.9.5)^33^ was used to perform a substructure search on the Enamine REAL Space (v04-2021). SMART patterns were generated for each scaffold identified during the exploration of the low-weight collection (Table S1). SpaceMACS queries generated up to 20 million compounds with the desired substructure and with 25 to 35 heavy atoms. The top 10 million drug-like molecules were then selected, following Lipinski rules and having <8 rotatable bonds, to generate the scaffold-focused libraries. Possible PAINS compounds were also discarded using the appropriate filters in MOE v2020.09, and after the generation of protonation and tautomerization states (Jchem v.20.21.0) and the appropriate stereoisomers and ring conformations, the combined number of molecular structures for the initial docking stage was ca. 35 million.

### Identification of the BRD4 (BD1) interaction hotspots by MDMix

The prepared BRD4 (BD1) receptor structure was employed for the mixed organic/aqueous solvent molecular dynamics simulations (MDMix). The protein was solvated with ethanol-water 1:4 and pyridine 1:20 truncated octahedral boxes. Then it was simulated for three replicates of 50 ns per solvation following the previously described standard protocol in pyMDMix^29^. The main interaction hotspots were identified from top occupancies (0.002 percentile) of the solvents, decomposed by interaction type.

### Virtual screening of the low molecular weight compounds

Initial binding modes for the low molecular weight compounds were then obtained by means of docking using rDock^34^. The cavity was defined as a 6 Å radius sphere centered at the position of the center of masses of the co-crystallized molecule 3-methyl-4phenyl-1,2-ozazol-5-amine, using the reference ligand protocol as implemented in the rDock software. Docking of the low molecular weight collection was performed, including two mandatory pharmacophoric restraints: namely, one H-bond acceptor was required at a distance of 2.9 ± 0.5 Å radius from the Nδ of Asn140, and one hydrophobic moiety was required at a distance of 3 ± 1 Å radius of the crystallographic water network^48^. The high-throughput VS (HTVS) setting was used during this stage, with up to 15 iterations of the genetic algorithm. A minimum threshold value of -12 points of interaction score (SCORE.INTER) was used to filter out compounds during the HTVS, yielding ca. 360000 high-scoring fragments. These fragments were characterized using the A1-A5 Chemical Checker signaturizers^32,49^, which include information related to 2D and 3D topological fingerprints, scaffolds, structural keys, and broad physicochemical properties. The obtained signatures were then clustered using the K-means algorithm with a fixed number of 2000 clusters, and the compound closest to the centroid of each cluster was further evaluated by single-point MM/GBSA calculations using Schrödinger’s Prime MM/GBSA tool^50^, retaining only those compounds with predictions of ΔG_bind_ lower than or equal than −30 kcal/mol for DUck simulations. In the last stage of the screening, DUck was used to rank ca. 970 fragments based on the W required to break the H-bond interaction between Nδ of Asn140 of BRD4 (BD1) and an H-bond acceptor in the ligand. The protein “chunk” was prepared manually selecting residues within 6 Å of Asn140 and included residues 81–89, 91–94, 97, 101, 104, 105, 131, 135–137, 139,140, 144–146, and 149 (numbering according to structure with PDBid 4LR6), as well as seven structural water molecules. For each molecule, DUck simulations were performed up to five times at 298 K and five times at 303 K, filtering out the compound earlier if W_QB_ was found below a threshold of 7 kcal/mol in any of the individual trajectories.

### Virtual screening of the scaffold-focused libraries

Before starting the virtual screening, ligands within each scaffold-focused library were aligned using their maximum common substructure (MCS) to the parent fragment, and atoms corresponding to the MCS were tethered, with a minimum translation and rotation threshold (<0.01 Å). The HTVS protocol of rDock was then used to obtain binding modes for each of the members of each library, using a protocol very similar to the one used in the screening of the low molecular weight collection, with the exception that the genetic algorithm of rDock was run for a maximum of 25 iterations, given a minimum score of −12 kJ/mol. Within each library, high-scoring compounds were again clustered using the A1-A5 Chemical Checker signaturizers and the K-means algorithm with a fixed number of 1000 clusters, and the compound closest to the centroid of each cluster was further evaluated by MM/GBSA. DUck was used to rank the top 500 compounds from each scaffold, using the same protocol and criteria used for the low molecular weight screening. A consensus ranking between DUck and MM/GBSA was used to select the 10 compounds from each scaffold-focused library to be tested experimentally.

### Protein expression and purification

A pET28 plasmid containing residues 44–168 of BRD4 (BD1), a 6xHis tag, a TEV protease cleavage site, and a kanamycin resistance gene was cloned in Rosetta D3 *E. Coli* competent cells. Transformed cells were cultured overnight in 10 mL of LB media supplied with kanamycin (50 μg/mL), then scaled to 1 L of LB medium supplied with kanamycin (50 μg/mL) and grown at 37 °C. Once the optical density (OD600) achieved 2.5, protein expression was induced with 0.4 mM isopropyl-β-thiogalactopyranoside (IPTG) overnight at 18 °C. Cells were harvested by centrifugation (8000 rpm, 4 °C, 30 min) and the pellet obtained was resuspended in 15 mL of Buffer A (50 mM Hepes, 150 mM NaCl, 30 mM Imidazole, 2 mM β-mercaptoethanol (pH 8.0) supplemented with Pierce protease inhibitor cocktail (ThermoFisher Scientific)) and lysed by sonication (1 min 40 s: 10 cycles × 10 s ON, 30 s OFF, *A* = 35%, *T* = 19 °C) followed by two consecutive centrifugations (8000 rpm, 4 °C, 30 min) to clarify the lysate. Supernatant was collected and filtered by 0.8 µm syringe filters, and BRD4 (BD1) was purified from it using an ÄKTA Start system (GE Healthcare, Uppsala, Sweden) by two consecutive immobilized metal ion affinity chromatography (IMAC) purifications. Specifically, the supernatant was applied to a 5 mL HisTrap HP column (Cytiva), washed with buffer A (50 mM Hepes, 150 mM NaCl, 30 mM Imidazole, 2 mM β-mercaptoethanol (pH 8.0)), and the bound protein was eluted with a linear gradient of buffer B (50 mM Hepes, 150 mM NaCl, 250 mM Imidazole, 2 mM β-mercaptoethanol (pH 8.0)). The eluted protein-containing fractions were pooled and concentrated. For protein to be used in SPR and TR-FRET experiments (*vide infra*), the purification procedure was terminated at this stage. For experiments that required prior His6 tag cleavage, Tev protease (Merck) was added to the protein fraction, and the mixture was dialyzed with buffer A (50 mM Hepes, 150 mM NaCl, 30 mM Imidazole, 2 mM β-mercaptoethanol (pH 8.0)) using a 7 kDa dialysis cassette (ThermoFisher Scientific). After cleavage, a second IMAC purification was performed as described, and the flow-through with the protein was collected. Finally, the mass and purity of the protein were verified by an SDS-Electrophoresis and mass spectrometry.

### Differential scanning fluorimetry

Thermal shift experiments were performed using a Light Cycler 480 II (Roche Applied Science) at the Genomics Service (CCiTUB). Compound screening was carried out in a 96-well plate containing a mixture of BRD4 (BD1) (2 µM), the fluorescence probe (SYPRO Orange - 1X), and ligand solution for a final volume of 25 µL. The DMSO concentration was kept at a maximum of 1% v/v in the final mixture. The experiment buffer used was 50 mM HEPES (pH 7.5), 150 mM NaCl. Compounds were screened at three different concentrations (50 µM, 10 µM, 1 µM) by triplicate (*n* = 3). In each experiment, (+)−JQ1 was tested at 10 µM (1% v/v DMSO) as a positive control. Negative controls were also added in the experiment (without ligand), consisting in protein and dye mixture and buffer and dye mixture. Excitation and emission filters for the SYPRO-Orange dye were set to 465 nm and 580 nm. To perform the experiments, the temperature was raised from 20 °C to 85 °C in 0.6 °C per minute steps. Data analysis was performed using the Light Cycler 480 software (Roche Applied Science). Only thermal shifts (ΔTm) of a magnitude at least twice the standard deviation (SD) of the average of Tm triplicates (*n* = 3) of the protein control were considered to indicate ligand binding.

### Surface plasmon resonance

All molecules were further assessed by Surface Plasmon Resonance (SPR) using a Biacore T200 SPR biosensor instrument (GE Healthcare, Uppsala, Sweden) at 25°C. His6-BRD4 (BD1) protein was immobilized on a NIHC1500M sensor chip (Xantec) via the non-covalent interaction between the nickel-treated chip surface and the His6 tag of the protein, followed by a standard covalent immobilization via amine coupling to avoid protein leaking during the different cycles. First, the preactivated and preconditioned NIHC1500M chip was treated with NTA loading buffer (5 mM NiCl2, 10 mM HEPES, 150 mM NaCl, 50 μM EDTA, 0.005% Tween-20) using one injection of 24 sec at 5 μL/min. Subsequently, the carboxymethyl dextran matrix was activated injecting a solution containing 0.1 M N-hydroxysuccinimide and 0.4 M 1-ethyl-3-(3- (dimethylamino)propyl) carbodiimide hydrochloride at a flow rate of 15 μL/min for 7 min. Once activated, the protein was immobilized, particularly oriented through the 6xHis tag using the affinity of the 6xHis to the coated Nickel on the chip surface. Protein immobilization was achieved after several injections of 4 μg/mL 6xHis-BRD4 (BD1) in 10 mM HEPES, 150 mM NaCl, 50 μM EDTA, 0.05% Tween-20 (pH 7.4) at a flow rate of 5 μL/min to achieve ~1600 RUs. The non-reacted but activated groups of the dextran matrix were deactivated by injection of 1 M ethanolamine hydrochloride for 7 min at a flow rate of 15 μL/min. The corresponding matrix activation and protein immobilization were performed using as a running buffer (RB) the following solution: 10 mM HEPES, 150 mM NaCl, 50 μM EDTA, 0.05% Tween-20 (pH 7.4).

Compounds were screened in randomized triplicate at 10 μM to discriminate between the positive and the negative binders. Compounds were prepared in a 100% DMSO stock solution at 100 mM and diluted with 1.01 × RB to achieve a final 1% (v/v) DMSO concentration. The flow rate used for the screening was 60 μL/min, and the ligand association and dissociation times were set at 60 s and 120 s, respectively. The flow rate and the association and dissociation times were maintained, and the compound titrations were analyzed by random duplicates (*n* = 2). The Biacore T200 Evaluation software was used for data analysis, correcting for nonspecific binding to the chip surface and for the baseline drift using the signals for a reference surface (where the immobilization procedure was carried out without proteins) to the signals obtained on the 6xHis-BRD4 (BD1) surface. Background noise was corrected by subtracting signals from blank injections from the compound signals. Sensorgrams as a function of concentration were used to obtain the steady state values, and the binding affinity was calculated by fitting the data to a single-site interaction model, fixing the R max according to the amount of protein immobilized on the chip surface.

### Time-resolved förster energy transfer (TR-FRET)

The HTRF TR-FRET competitive binding assay was used to measure the disruption of the interaction between BRD4 (BD1) and an acetylated [Lys(Ac)5/8/12/16]-Histone H4 (1-21)-GGK(Biotin). TR-FRET experiments were performed using a CLARIOstar (BMG Labtech) plate reader using the homogeneous time-resolved fluorescence module (excitation, 337 nm with 200 flashes; emission, 620 and 665 nm). Compound screening was carried out in 384-well, white, round-bottom, small-volume plates (CORNING) in a final volume of 20 µL of 50 mM HEPES (pH 7.4), 50 mM NaCl, 400 mM KF, 0.5 mM CHAPS, and 0.05% BSA. The His-tagged BRD4 (BD1) (100 nM) was first mixed with the biotin-labeled histone peptide (Eurogentec) (200 nM) and incubated for 10 min. Then, the labeled anti-His6-XL665 antibody (CisBio) (10 nM) was added to the mixture and incubated for 15 min, before the Eu3+ cryptate-conjugated streptavidin (2 nM) (CisBio) was added and incubated for 15 min. All incubations were performed at room temperature. Compounds were titrated (from 0.1 nM to 10 µM) by duplicate (*n* = 2), and the response at each point was averaged to determine the IC_50_ for each compound. Titration of (+)−JQ1 was used as a positive control for each experiment. The 665 nm/620 nm ratio was converted to % Normalized HTRF ratio (top signal equals 100% and bottom signal equals 0%) for each compound, and the IC_50_ was determined using Hill’s equation with a standard Hill slope using GraphPad Prism software.

### X-ray crystallography

BRD4 (14 mg/ml) crystallized readily in multiple positions of the Morpheus screen. Subsequently, BRD4 was saturated with ligands, allowing up to a 20% v/v DMSO for co-crystallization experiments. Crystals were grown using the sitting-drop technique at 19 °C. Grown crystals were retrieved with a nylon and soaked in the crystallization buffer saturated with ligand at 20 v/v% (DMSO) for 1 h to be finally flash-frozen in liquid nitrogen for X-ray data collection.

### Data collection and structure refinement

Data sets were collected at the P13 EMBL beam line of the Petra III synchrotron. Data reduction and scaling were performed directly using AutoproC^51^. The structure was solved by molecular replacement using Phaser^52^ from the Phenix suite^53,54^. Subsequently, model building and structure refinement used iterative rounds with Coot^55^ combined with refinement in Phenix. Refine. A final round of refinement was done with the PDB-redo^56,57^. Summary of the statistics for data collection and model building is provided in Table S4.

### Reporting summary

Further information on research design is available in the Nature Portfolio Reporting Summary linked to this article.

## Supplementary information

Supplementary information

Description of Additional Supplementary Files

Supplementary Data 1

Supplementary Data 2

Supplementary Data 3

Supplementary Data 4

Reporting Summary

## Data Availability

The protein structures used for the structural mapping are available in the Worldwide Protein Data Bank (WWPDB.org), under the following PDB identifiers: PDB:9HT2 (**Ligand 92**), PDB:9HT1 (**Ligand 94**), PDB:9HT0 (**Ligand 50**). All other data are available within the main text or the Extended Data. Raw data is available in Supplementary Information. Source data are provided in the Supplementary_Data file attached to this paper.

## Code availability

No new code was generated for this study. The analyses were based on existing published algorithms.
