# Supplementary material for: A bottom-up approach to find lead compounds in expansive chemical spaces
Source: Commun Chem. 2025 Aug 1;8:225. doi: 10.1038/s42004-025-01610-2 (PMC12316891; doi:10.1038/s42004-025-01610-2)
Supplement: Supplementary file 1 — Supplementary information [file 42004_2025_1610_MOESM1_ESM.docx]

Supporting Information

A bottom-up approach to find lead compounds in expansive chemical spaces

Álvaro Serrano-Morrás,^1,2Φ^ Andrea Bertran-Mostazo,^1,2Φ^ Marina Miñarro-Lleonar,^1,2^ Arnau Comajuncosa-Creus,^3^ Adrià Cabello,^1^ Carme Labranya,^1^ Carmen Escudero^4^, Tian Tian^4^, Inna Khutorianska^5^, Dmytro S. Radchenko^5^, Yurii S. Moroz^5,6,7^, Lucas Defelipe^8,9^, David Ruiz-Carrillo,^8,9^ Maria Garcia-Alai,^8,9^ Robert Schmidt^10^, Matthias Rarey^10^, Patrick Aloy,^3,11^ Carles Galdeano,^1,2^ Jordi Juárez-Jiménez,^1,12*^ Xavier Barril^1,2,11,12,*^

*^1^Unitat de Fisicoquímica, Departament de Farmàcia i Tecnologia Farmacéutica, i Fisicoquímica. Facultat de Farmàcia I Ciències de l’Alimentació. Universitat de Barcelona (UB). Av. Joan XXIII, 27-31, 08028 Barcelona, Spain.*

*^2^Institut de Biomedicina, Facultat de Biologia, Universitat de Barcelona (UB), Av. Diagonal, 643, 08028, Barcelona, Spain*

*^3^ Institut de Recerca Biomèdica (IRB Barcelona) and Barcelona Institute of Science and Technology (BIST). c/ Baldiri i Reixac, 10-12, 08028 Barcelona, Catalonia, Spain.*

*^4^ Upper Gastrointestinal and Endocrine Tumor Group, Vall d'Hebron Institute of Oncology (VHIO), Barcelona, Spain.*

*^5^ Enamine Ltd., 78 Winston Churchill Street, Kyїv 02094, Ukraine*

*^6^ Chemspace LLC, 85 Winston Churchill Street, Kyїv 02094, Ukraine*

*^7^ Taras Shevchenko National University of Kyiv, Volodymyrska Street 60, Kyїv 01601, Ukraine*

*^8^ European Molecular Biology Laboratory, Building 25a, DESY, Hamburg, 22607, Germany*

*^9^ Centre for Structural Systems Biology (CSSB), Building 15, DESY, Hamburg, 22607, Germany*

*^10^ University of Hamburg, ZBH – Center for Bioinformatics, Albert-Einstein-Ring 8-10, 22761 Hamburg, Germany*

*^11^Catalan Institution for Research and Advanced Studies (ICREA), Pg. Lluís Companys, 23 08010, Barcelona, Spain.*

*^12^ Institut de Química Teòrica i Computacional (IQTC), Facultat de Química i Física, Universitat de Barcelona (UB). C. Martí i Franqués, 1, 08028, Barcelona, Spain.*

^Φ^ These authors contributed equally to this work.

^β^ Current address: BioSolveIT GmbH - An der Ziegelei 79 - 53757 St.Augustin - Germany.

Table of contents

Figure S1. Energy surface and interaction hotspots of the MDMix ethanol probes.

Figure S2. Selection criteria for the exhaustive fragment-based screening.

Figure S3. Relationship between the enumerated molecules during the scaffold search and the computational cost.

Figure S4. Representation of the reference scaffolds’ binding mode.

Figure S5. Distribution of rDock scores for the best pose and conformation of each compound separated by scaffold.

Figure S6. Distribution of ΔGbind calculated with MM/GBSA.

Figure S7. Distribution of WQB values for the cluster representatives.

Figure S8. Example of assessment of the clustering quality using IBET scaffold-focused library.

Figure S9. Structure of the compounds bought from Enamine Real.

Figure S10. DSF profiles of some compounds tested.

Figure S11. Quality control SDS-Page and/or MALDI-TOF protein purification BRD4 (BD1).

Table S1. SMARTS queries for the construction of the scaffold-focused libraries

Table S2. Number of compounds per scaffold per step during the pipeline

Table S3. Calculation wall clock time per compound per step during the pipeline

Table S4. Data collection and refinement statistics of the crystal strucures


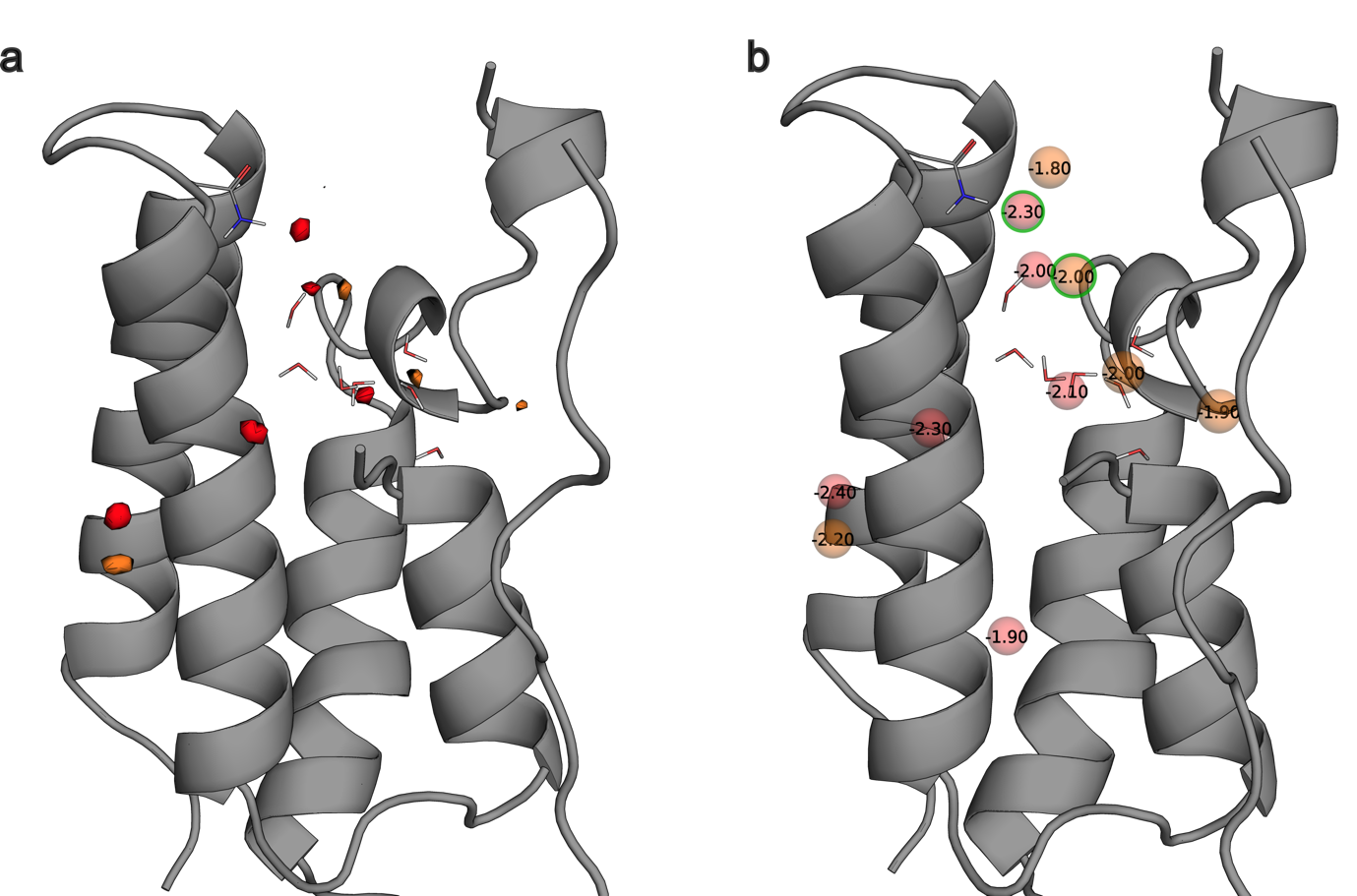


**Figure S1.** Energy surface and interaction hotspots of the MDMix ethanol probes. a)The red surfaces correspond to the alcohol group and the orange surfaces to the terminal carbon at a -2 kcal/mol threshold. b) Interaction hotspots, labelled with the energy derived from the Boltzmann average of the interaction energy maps around the highest energy points in the grid. The two hotspots highlighted in green correspond to those selected as pharmacophoric restrictions for the virtual screening.


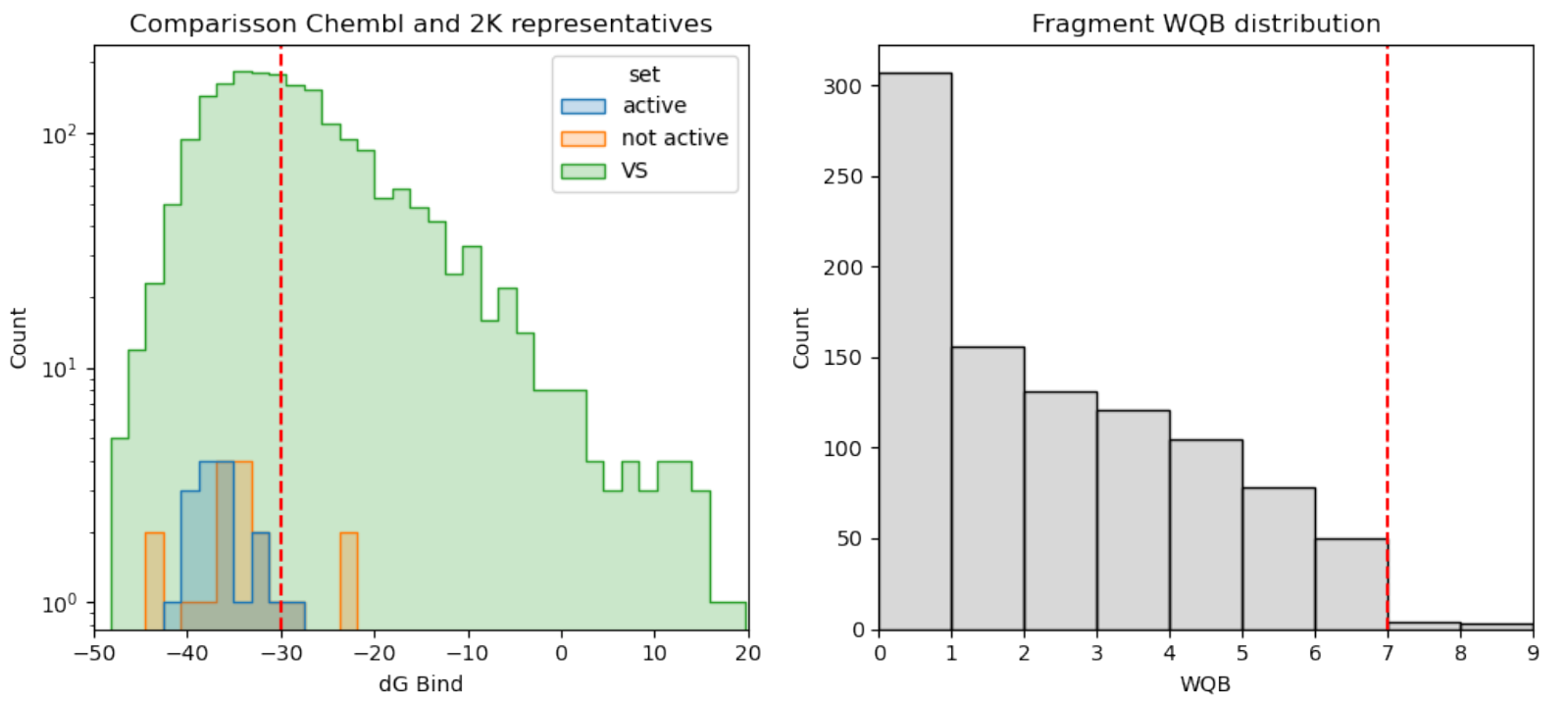


**Figure S2.** Selection criteria for the exhaustive fragment-based screening. On the left, the distribution of ∆Gbind for the active Chembl set, the non-active CHEMBL set and the fragments obtained in the VS. The red line represents the chosen threshold value. On the right, the distribution of W_QB_ values for the VS fragments and the red line represents the threshold value.


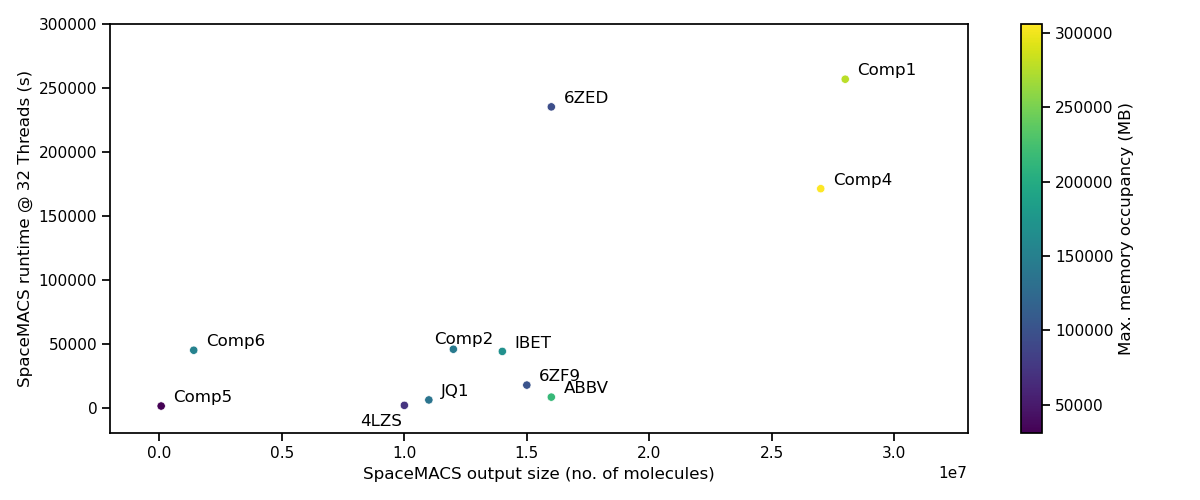


**Figure S3.** Relationship between the enumerated molecules during the scaffold search and the computational cost. The colorbar refers to the maximum RAM memory allocated during the query and the y-axis corresponds to the wallclock time registered during the calculation, including writing the output.


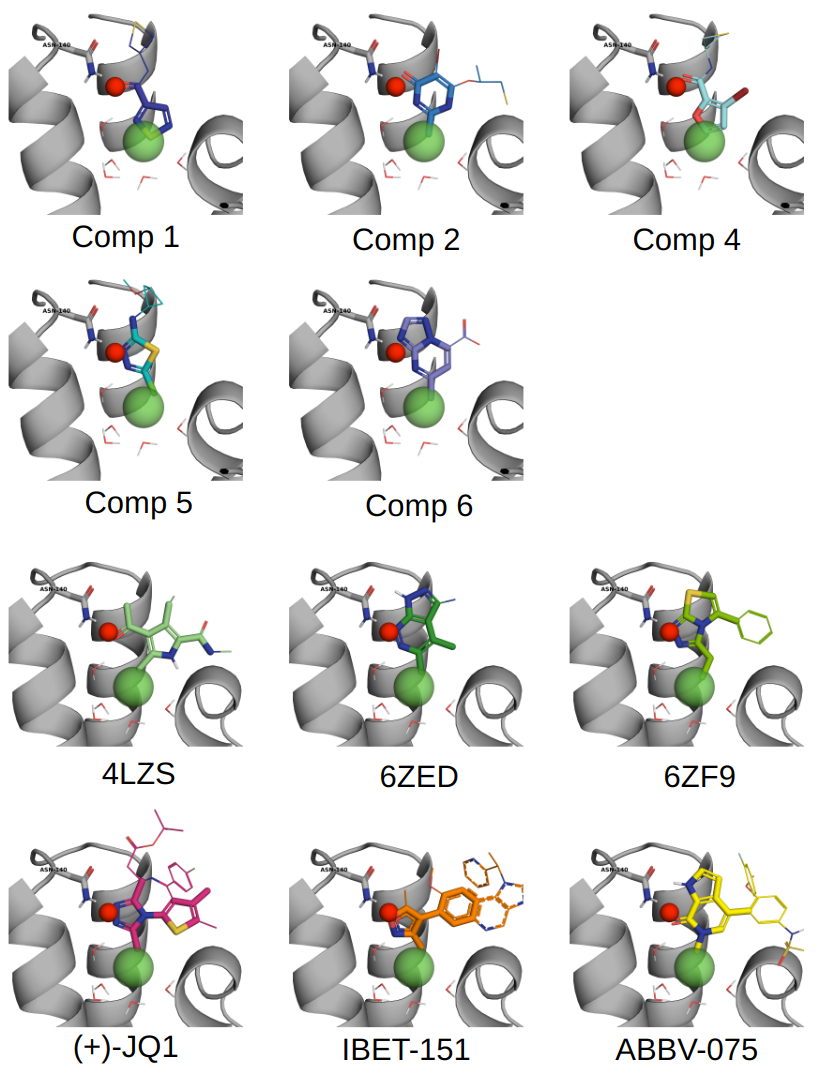


**Figure S4.** Representation of the reference scaffolds’ binding mode. The structure of the reference virtual hits, experimental fragments and drug candidates is represented in lines. The scaffolds used as query and tethered on their analogs are represented in sticks. The H-bond acceptor and hydrophobic pharmacophoric restraints are represented as red and orange spheres respectively.


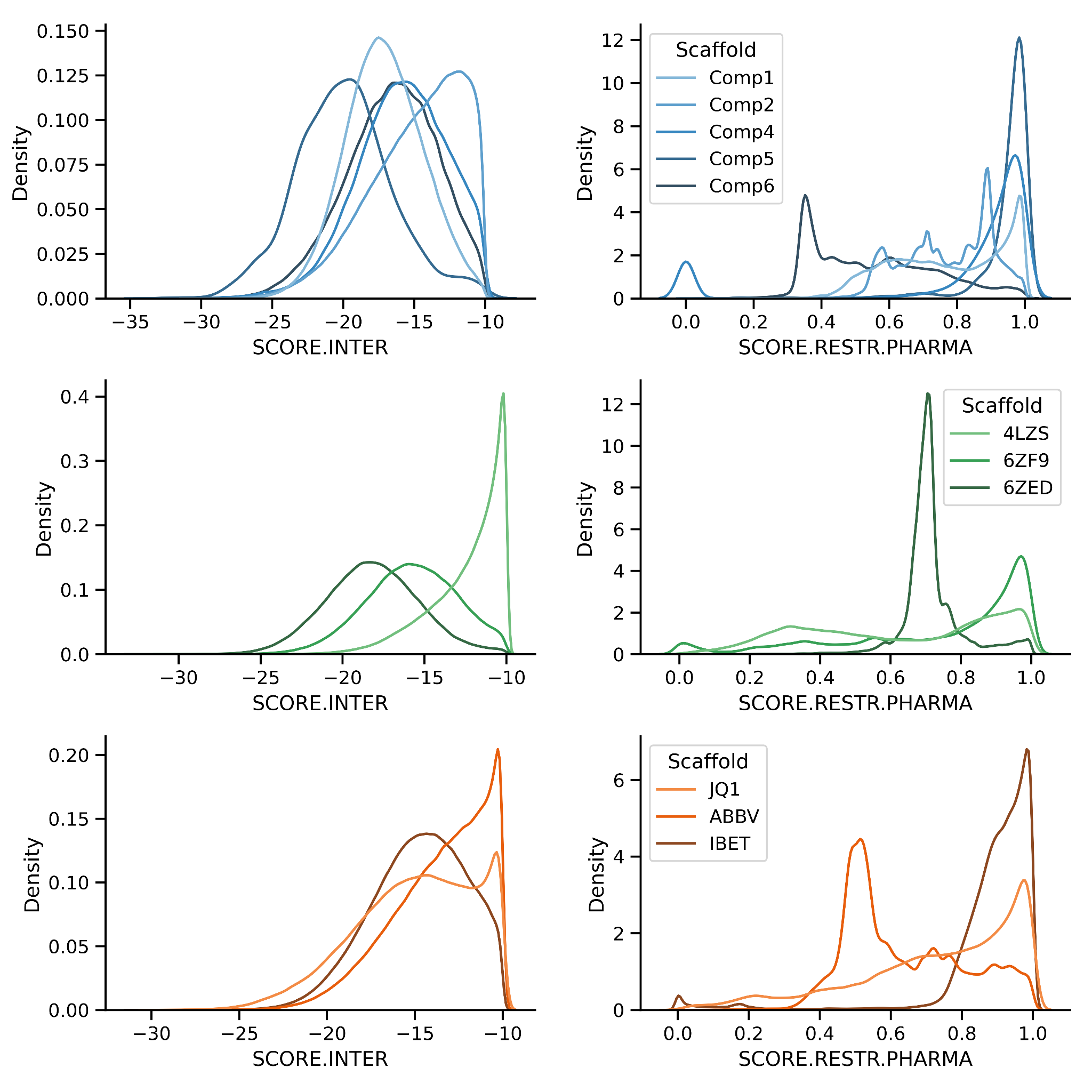


**Figure S5.** Distribution of rDock scores for the best pose and conformation of each compound separated by scaffold. The HTVS filter limits the output binding poses with SCORE.INTER and SCORE.RESTR.PHARMA lower than -10 and 1 respectively.


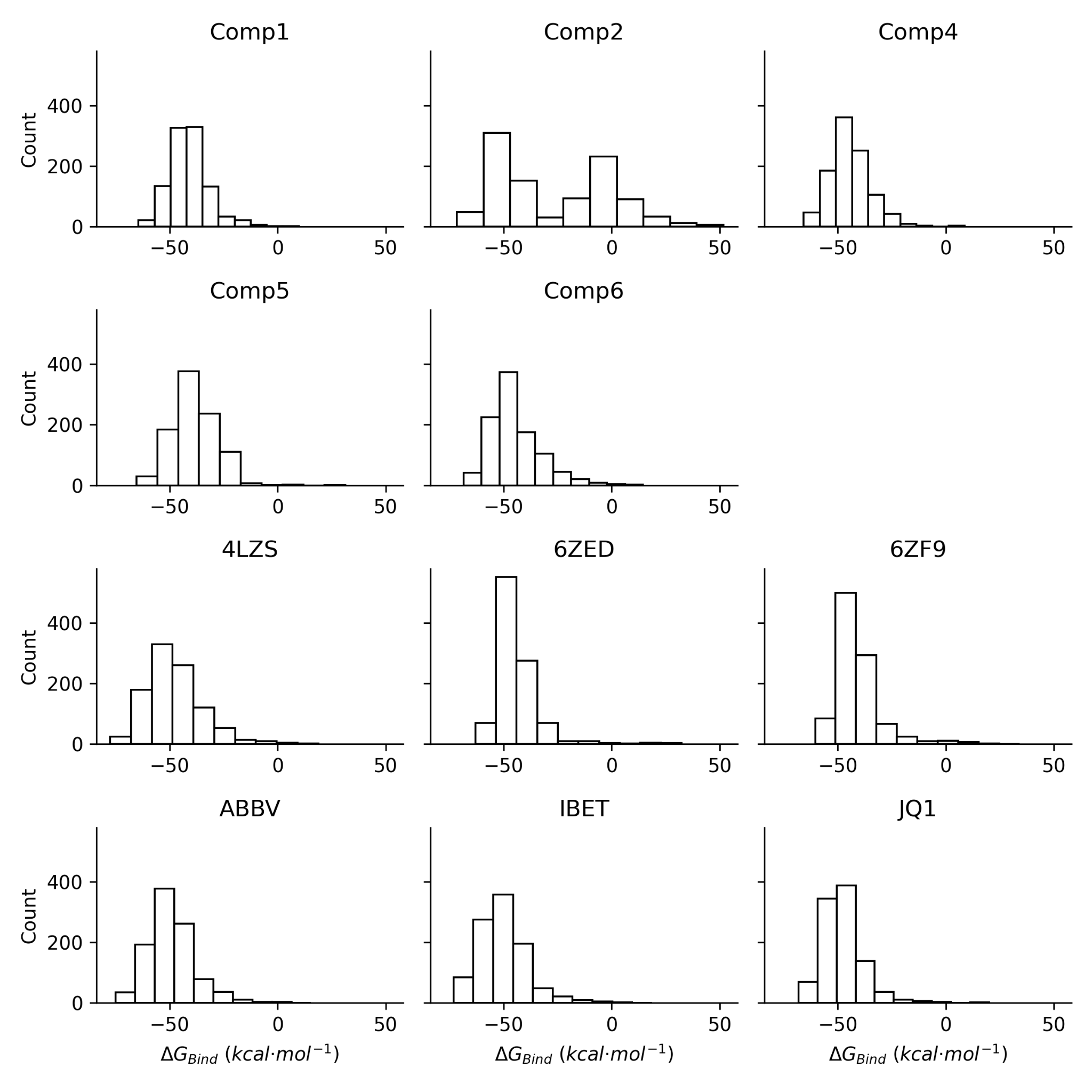


**Figure S6.** Distribution of ΔG_bind_ calculated with MM/GBSA. The ΔG_bind_ correspond to single-point calculations taking into account the ligands’ strain.


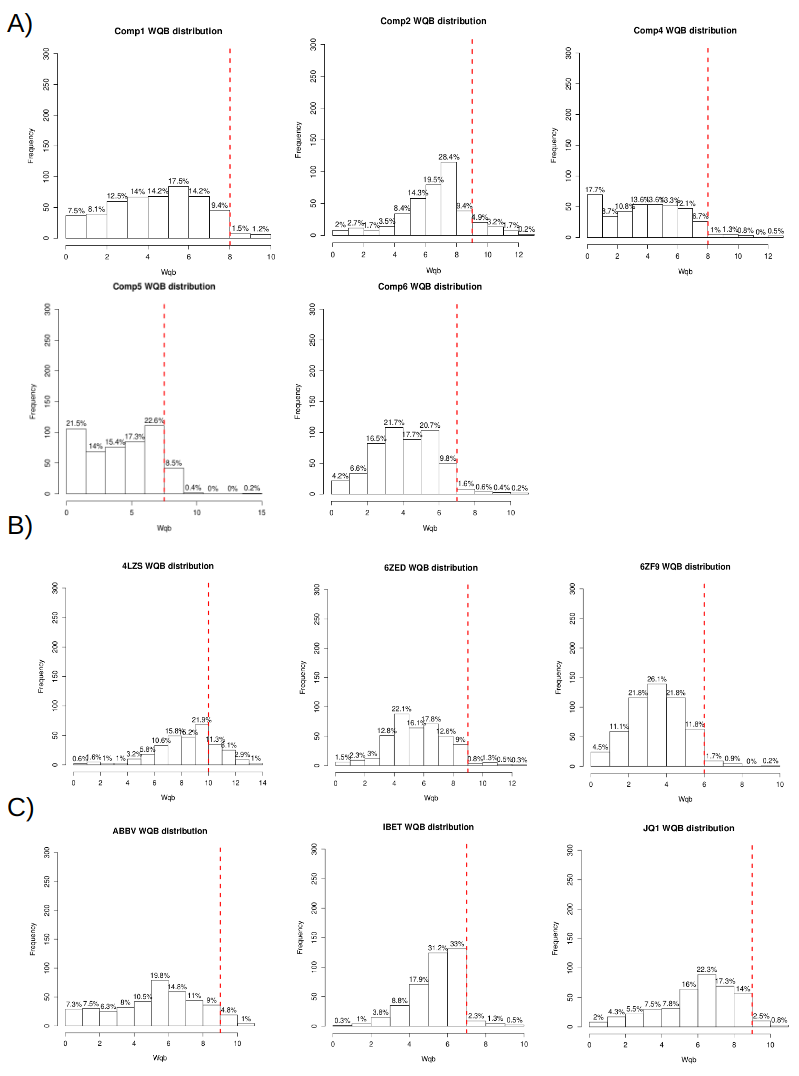


**Figure S7.** Distribution of W_QB_ values for the cluster representatives. A, B and C correspond to hits derived from the virtual fragments, experimental fragments and drug scaffolds’ respectively. The red dotted lines represent the selected W_QB_ threshold values.


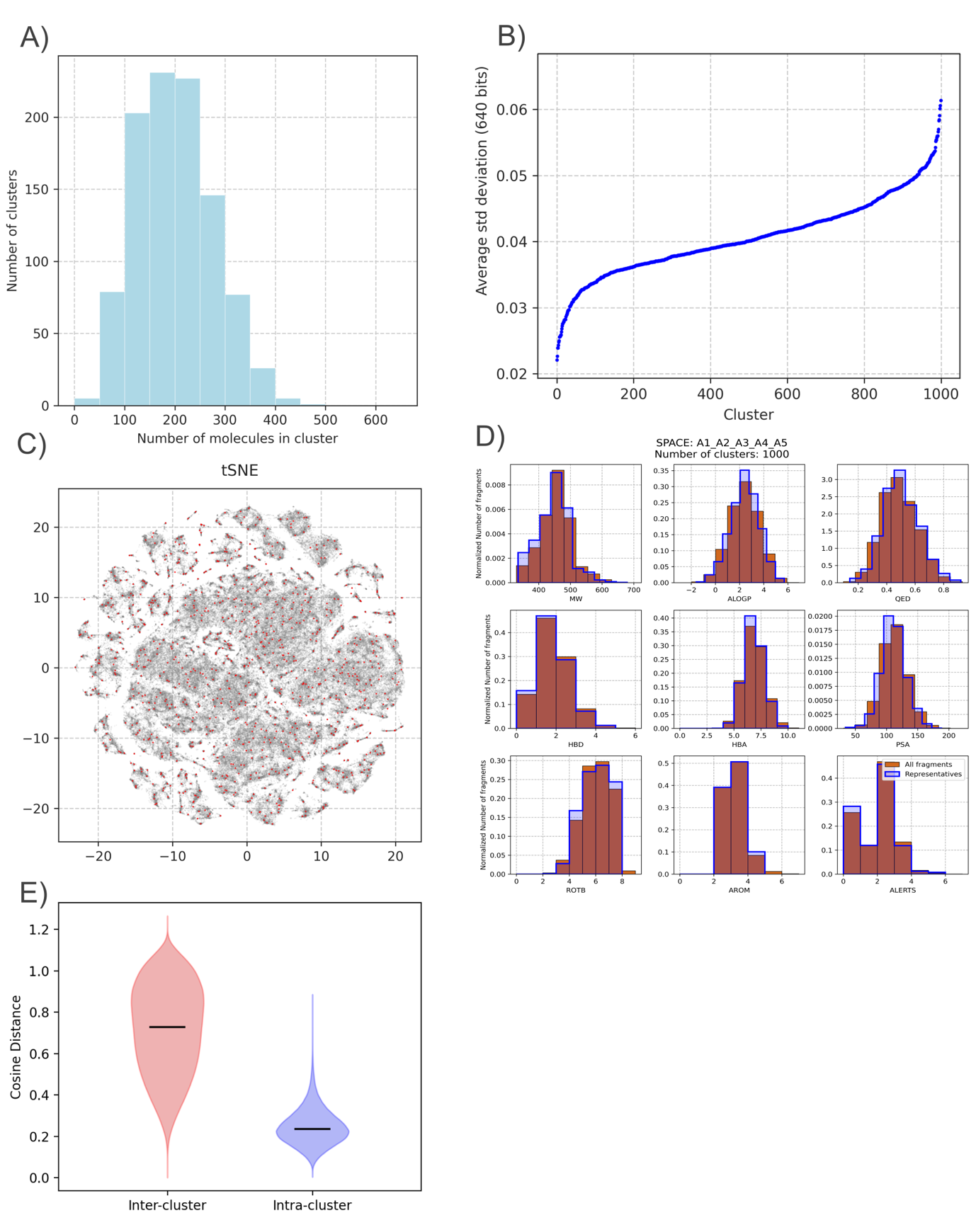


**Figure S8.** Example of assessment of the clustering quality using IBET scaffold-focused library. A) Shows the distribution of compounds per cluster. B) corresponds to the standard deviation of similarities within each clustered. C) Is a tSNE dimensionality reduction with the red dots representing the cluster representatives. D) Shows the overlap in physicochemical properties of the representatives and the rest of the library. E) Shows the distribution of inter- and intra-cluster cosine distances between each pair of compounds.





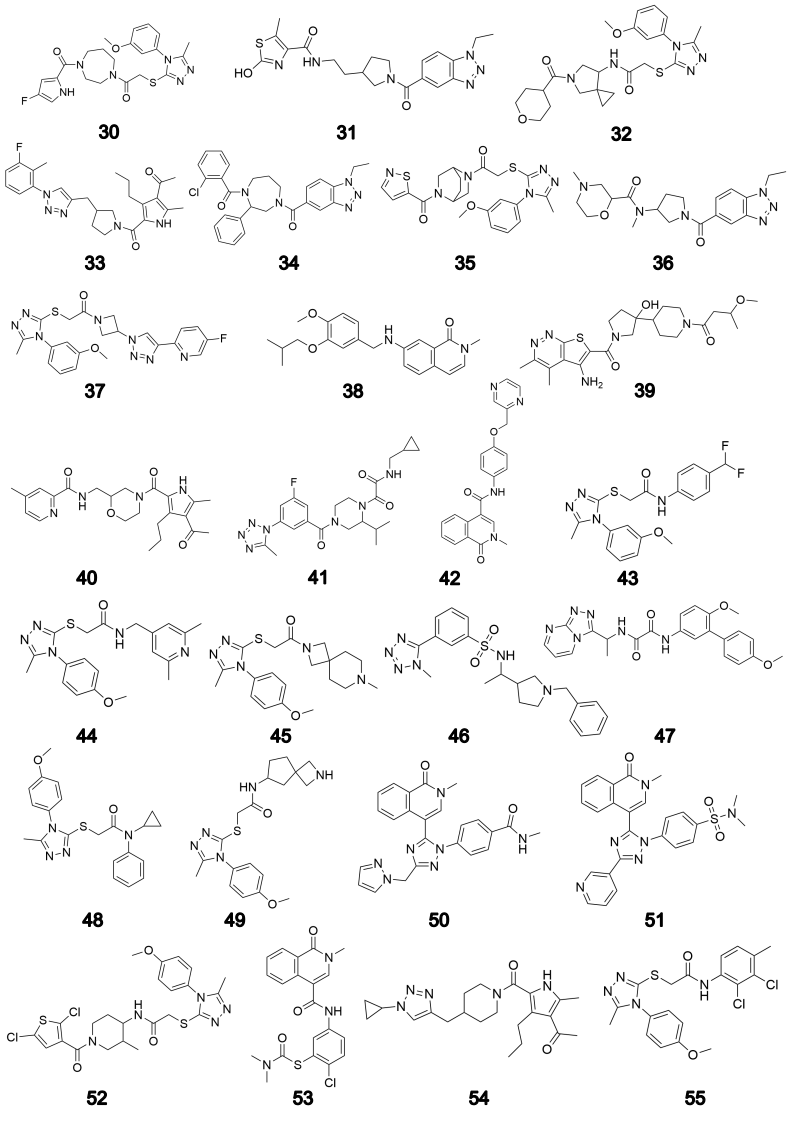

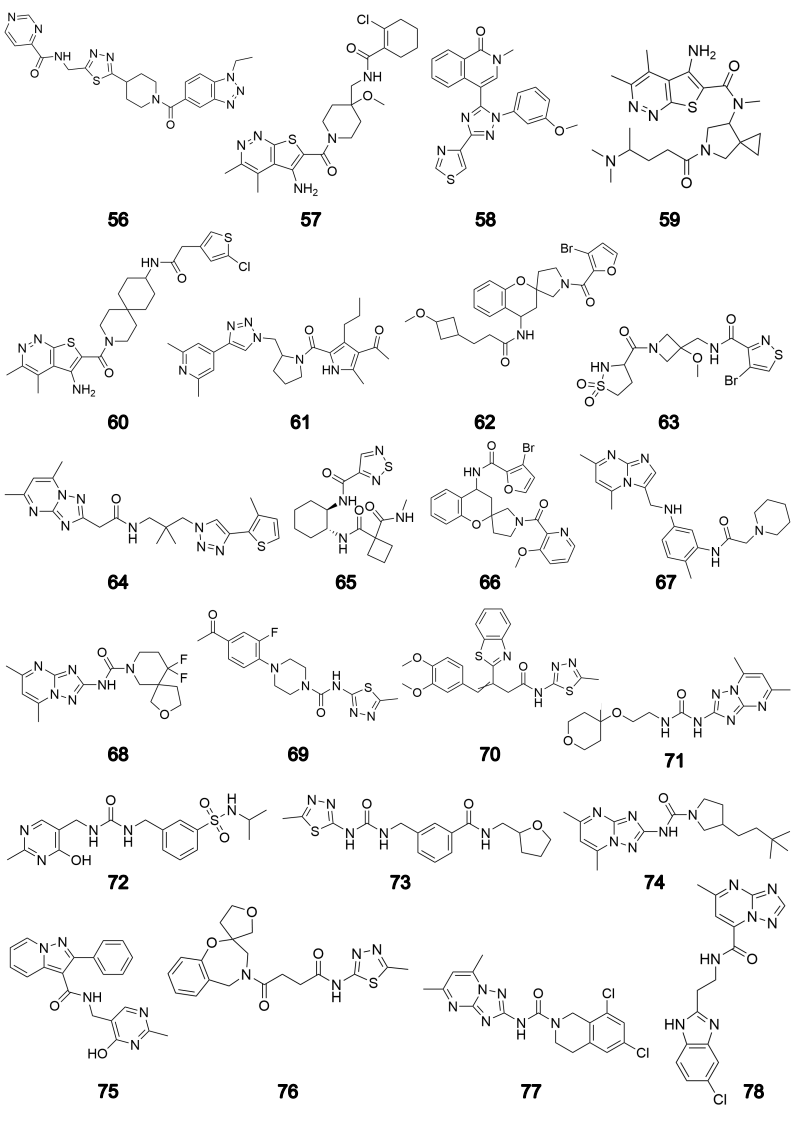

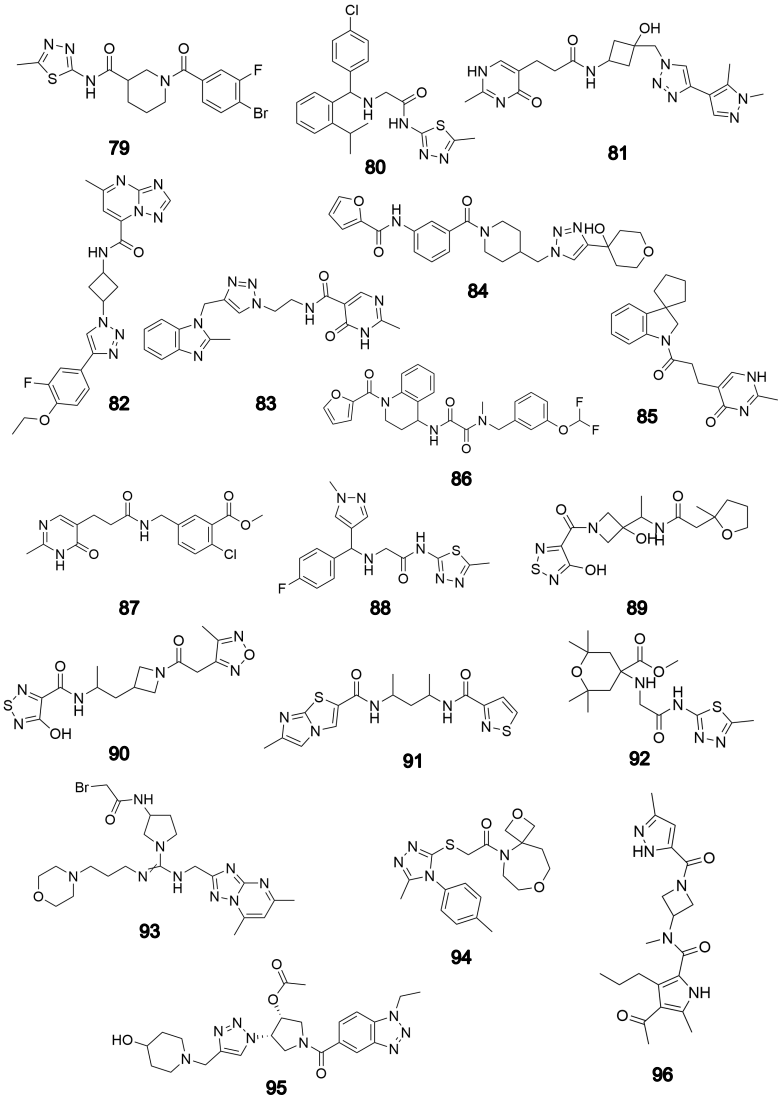


**Figure S9.** Structure of the compounds bought from Enamine Real.


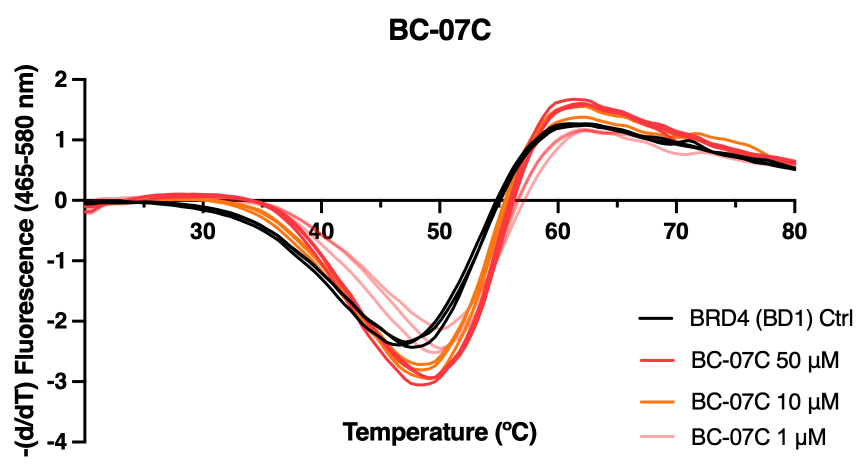


**Compound 50**


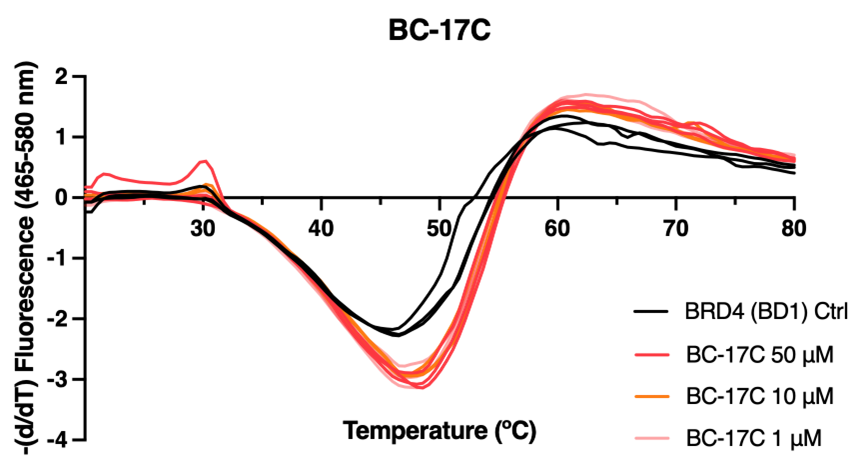


**Compound 94**


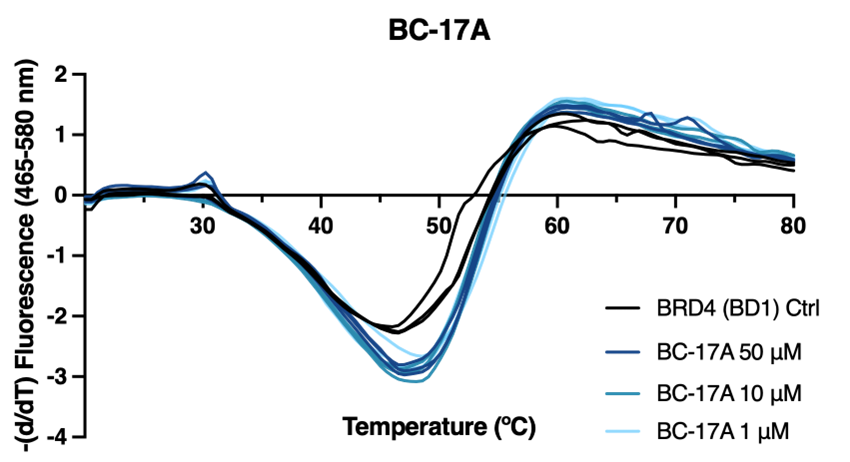


**Compound 92**

**Figure S10**. DSF profiles of some compounds tested.


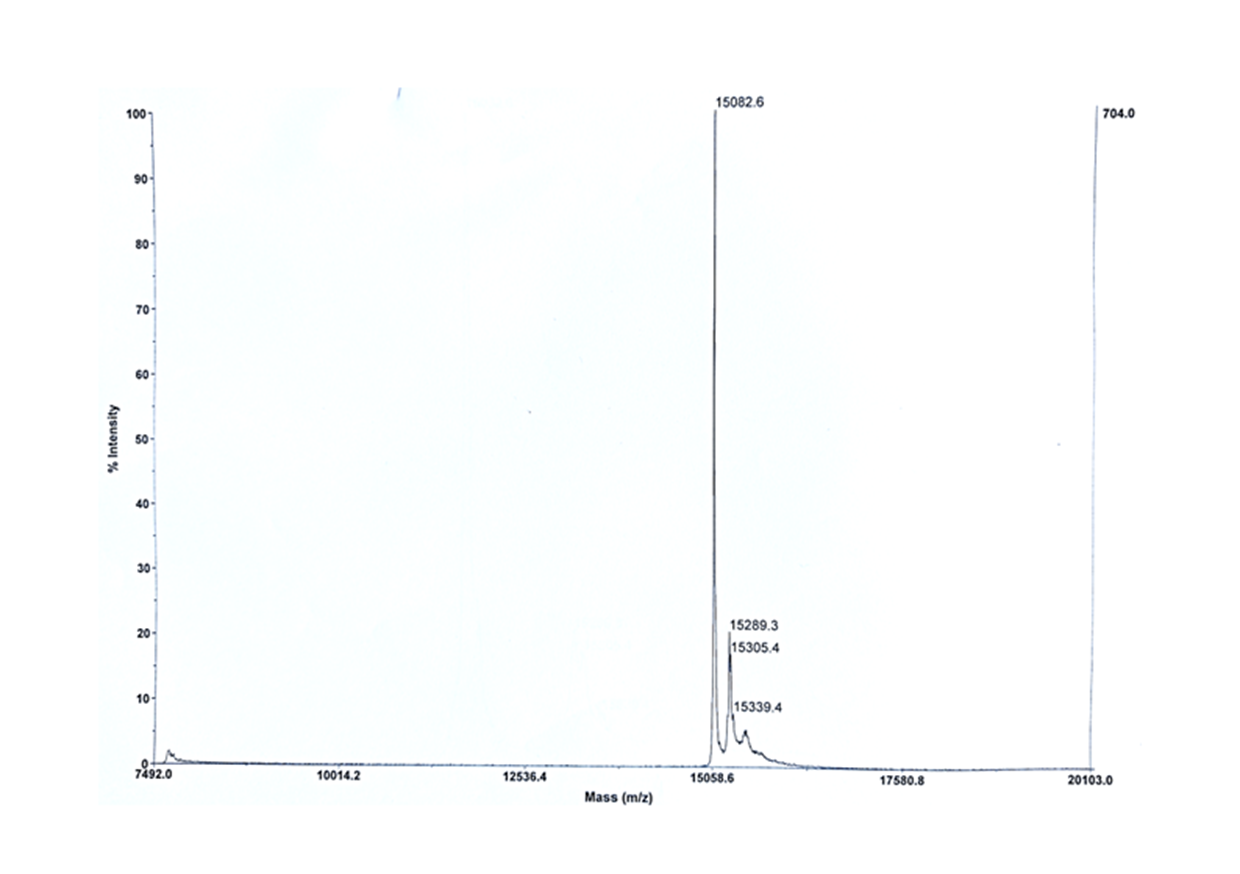

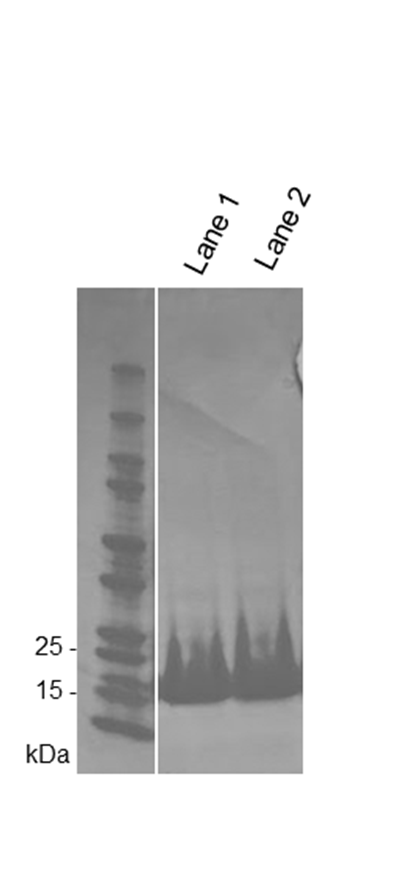


**Figure S11.** Quality control SDS-Page and/or MALDI-TOF protein purification BRD4 (BD1).

**Table S1.** SMARTS queries for the construction of the scaffold-focused libraries

| System | | SMARTS query |
| --- | --- | --- |
| Drug Candidates | ABBV-075 | [CH3]a1aa([R])a2aa[nH]a2c1=O |
|  | IBET151 | a1[nh0,o;D2][nh0,o;D2]a([CH3])a1(a1aaaaa1) |
|  | JQ1 | [CH3]a1[nh0;D2][nh0;D2]a(C)a1c2scaa2C |
| Experimental Fragment Hits | 4LZS | a1a([*][#7;D2;h1])[nH]a(C)a1C([*])=O |
|  | 6ZED | [CH3]a1[nh0;D2][nh0;D2]a2[nH]aaa2a1[*] |
|  | 6ZF9 | [CH3][*]a1[nh0;D2][nh0;D2]a2saa([R])a12 |
| Virtual Fragment Hits | Comp1 | [nH0]1saac1C(=O) |
|  | Comp2 | O=c1[nD2]c([CH3])[nD2]cc1 |
|  | Comp4 | o1[cH][cH]cc1C=O |
|  | Comp5 | n1nc([D1])sc1[NH] |
|  | Comp6 | [C,Cl;D1]c1aaa2aa[nD2;H0]a2[nD2;H0]1 |

**Table S2.** Number of compounds per scaffold per step during the pipeline (i.e. scaffold search, drug-like, 3D conformers, docking, post-docking, selected).

| Scenario / Scaffold | | SpaceMACS output | druglike conformers | Docking output | WQB threshold (kcal·mol^-1^) | DUck output | Compounds synthesized |
| --- | --- | --- | --- | --- | --- | --- | --- |
| Virtual Fragment hits | Comp1 | 2.8·10^7^ | 1.6·10^7^ | 2.7·10^6^ | 8 | 13 | 5 |
|  | Comp2 | 1.2·10^7^ | 2.7·10^7^ | 3.8·10^6^ | 9 | 41 | 6 |
|  | Comp4 | 2.7·10^7^ | 1.6·10^7^ | 2.2·10^5^ | 8 | 14 | 4 |
|  | Comp5 | 7·10^4^ | 3·10^5^ | 2.4·10^3^ | 8 | 13 | 8 |
|  | Comp6 | 1.4·10^6^ | 2.8·10^6^ | 5.3·10^5^ | 7 | 12 | 9 |
| Exp. Fragment hits | 4LZS | 10^7^ | 3.3·10^7^ | 10^6^ | 10 | 72 | 10 |
|  | 6ZED | 1.6·10^7^ | 3.6·10^7^ | 8.5·10^5^ | 9 | 11 | 6 |
|  | 6ZF9 | 1.5·10^7^ | 2.7·10^7^ | 7.3·10^5^ | 6 | 15 | 8 |
| Drug candidates | JQ1 | 1.1·10^7^ | 2.6·10^7^ | 5·10^5^ | 9 | 13 | 9 |
|  | IBET-151 | 1.4·10^7^ | 2.8·10^7^ | 2.8·10^6^ | 7 | 16 | 10 |
|  | ABBV-075 | 1.6·10^7^ | 2.9·10^7^ | 1.6·10^6^ | 9 | 23 | 10 |

**Table S3.** Calculation wallclock time per compound per step during the pipeline (i.e. scaffold search, drug-like, 3D conformers, docking, post-docking, selected). The accumulated wallclock time per scenario and step corresponds to single thread processes unless specified in parentheses.

|  | Scaffold search Query (SpaceMACS) | Library preparation & filtering | Docking | Clustering | MM/GBSA | DUck |
| --- | --- | --- | --- | --- | --- | --- |
| cpuseconds per compound | 0.15 | 0.12 | 7.5 | 0.036 | 96 | 1790±511 |
| Total Wallclock Scenario 1(s) | 5.20·10^5^  (@32 CPUs) | 5.36·10^6^ | 4.77·10^8^ | 3.8·10^4^ | 4.8·10^5^ | 8.35·10^5^  (@ GPU) |
| Total Wallclock Scenario 2(s) | 1.01·10^5^  (@32 CPUs) | 9.86·10^6^ | 1.38·10^9^ | 4.4·10^4^ | 5.8·10^5^ | 7.63·10^5^  (@ GPU) |

**Table S4.** X-Ray crystallographic quality parameters.

|  | LIG92 | LIG94 | LIG50 |
| --- | --- | --- | --- |
| PDB ID | 9HT2 | 9HT1 | 9HT0 |
| Resolution range (Å) | 26.92 - 1.42 (1.471 - 1.42) | 42.1 - 1.94 (2.009 - 1.94) | 38.82 - 1.33 (1.378 - 1.33) |
| Space group | P 1 21 1 | P 21 21 21 | P 21 21 21 |
| Unit cell | 30.353 39.553 55.861 90 105.492 90 | 41.989 79.035 84.194 90 90 90 | 36.1 44.587 78.907 90 90 90 |
| Total reflections | 165859 (15951) | 282070 (28746) | 381979 (37808) |
| Unique reflections | 23321 (2271) | 21437 (2127) | 29986 (2939) |
| Multiplicity | 7.1 (7.0) | 13.2 (13.5) | 12.7 (12.9) |
| Completeness (%) | 95.93 (93.40) | 99.95 (100.00) | 99.87 (99.46) |
| Mean I/sigma(I) | 13.07 (2.60) | 12.97 (2.40) | 18.97 (2.62) |
| Wilson B-factor | 17.61 | 25.00 | 16.56 |
| R-merge | 0.07624 (0.6479) | 0.142 (1.099) | 0.06058 (0.8042) |
| R-meas | 0.08237 (0.6998) | 0.1478 (1.142) | 0.06315 (0.837) |
| R-pim | 0.03085 (0.262) | 0.04064 (0.3088) | 0.01762 (0.2289) |
| CC1/2 | 0.998 (0.838) | 0.998 (0.778) | 0.999 (0.913) |
| CC* | 0.999 (0.955) | 0.999 (0.936) | 1 (0.977) |
| Reflections used in refinement | 23311 (2264) | 21429 (2127) | 29970 (2938) |
| Reflections used for R-free | 1210 (126) | 1033 (85) | 1479 (133) |
| R-work | 0.1717 (0.3019) | 0.1778 (0.2620) | 0.2246 (0.3905) |
| R-free | 0.1863 (0.3416) | 0.2216 (0.2854) | 0.2474 (0.3939) |
| CC(work) | 0.953 (0.724) | 0.948 (0.819) | 0.940 (0.468) |
| CC(free) | 0.918 (0.545) | 0.949 (0.755) | 0.921 (0.425) |
| Number of non-hydrogen atoms | 1254 | 2362 | 1246 |
| macromolecules | 1062 | 2124 | 1062 |
| ligands | 36 | 46 | 33 |
| solvent | 156 | 192 | 151 |
| Protein residues | 127 | 254 | 127 |
| RMS(bonds - Å) | 0.009 | 0.014 | 0.008 |
| RMS(angles – deg.) | 1.19 | 1.41 | 1.11 |
| Ramachandran favored (%) | 98.40 | 99.20 | 99.20 |
| Ramachandran allowed (%) | 1.60 | 0.80 | 0.80 |
| Ramachandran outliers (%) | 0.00 | 0.00 | 0.00 |
| Rotamer outliers (%) | 0.00 | 0.83 | 0.83 |
| Clashscore | 1.84 | 1.86 | 0.93 |
| Average B-factor (Å^2^) | 14.94 | 20.55 | 15.25 |
| Macromolecules (Å^2^) | 11.65 | 18.52 | 12.85 |
| Ligands (Å^2^) | 40.39 | 53.95 | 18.95 |
| Solvent (Å^2^) | 31.44 | 34.98 | 31.32 |
| Number of TLS groups | 1 | 14 | 7 |
