# Supplementary material for: A bottom-up approach to find lead compounds in expansive chemical spaces
Source: Commun Chem. 2025 Aug 1;8:225. doi: 10.1038/s42004-025-01610-2 (PMC12316891; doi:10.1038/s42004-025-01610-2)
Supplement: Supplementary file 2 — Description of Additional Supplementary Files [file 42004_2025_1610_MOESM2_ESM.pdf]

Description of Additional Supplementary Files

File name: Supplementary Data 1

Description: Raw data for the DSF, SPR and TR-FRET measurements

File name: Supplementary Data 2

Description: Deposited PDB structure of BRD4 bound to compound 92

File name: Supplementary Data 3

Description: Deposited PDB structure of BRD4 bound to compound 94

File name: Supplementary Data 4

Description: Deposited PDB structure of BRD4 bound to compound 50
